# The diversity of neuronal phenotypes in rodent and human autonomic ganglia

**DOI:** 10.1007/s00441-020-03279-6

**Published:** 2020-09-15

**Authors:** Uwe Ernsberger, Thomas Deller, Hermann Rohrer

**Affiliations:** Institute of Clinical Neuroanatomy, Dr. Senckenberg Anatomy, Neuroscience Center, Goethe University, Theodor-Stern-Kai 7, 60590 Frankfurt/M, Germany

**Keywords:** Sympathetic, Parasympathetic, Pelvic, Neurotransmitter, Neuron

## Abstract

Selective sympathetic and parasympathetic pathways that act on target organs represent the terminal actors in the neurobiology of homeostasis and often become compromised during a range of neurodegenerative and traumatic disorders. Here, we delineate several neurotransmitter and neuromodulator phenotypes found in diverse parasympathetic and sympathetic ganglia in humans and rodent species. The comparative approach reveals evolutionarily conserved and non-conserved phenotypic marker constellations. A developmental analysis examining the acquisition of selected neurotransmitter properties has provided a detailed, but still incomplete, understanding of the origins of a set of noradrenergic and cholinergic sympathetic neuron populations, found in the cervical and trunk region. A corresponding analysis examining cholinergic and nitrergic parasympathetic neurons in the head, and a range of pelvic neuron populations, with noradrenergic, cholinergic, nitrergic, and mixed transmitter phenotypes, remains open. Of particular interest are the molecular mechanisms and nuclear processes that are responsible for the correlated expression of the various genes required to achieve the noradrenergic phenotype, the segregation of cholinergic locus gene expression, and the regulation of genes that are necessary to generate a nitrergic phenotype. Unraveling the neuron population-specific expression of adhesion molecules, which are involved in axonal outgrowth, pathway selection, and synaptic organization, will advance the study of target-selective autonomic pathway generation.

## Introduction

The autonomic nervous system (ANS) in mammals functions to ensure the maintenance of body homeostasis under highly variable conditions, which allows mammals to access remarkably diverse environments, such as those experienced by diving animals (Scholander 1940; McCulloch 2012) and animals that roam desert habitats (Ouajd and Kamel 2009). The disruption of proper ANS functions in humans, associated with disease processes or trauma, can result in a wide range of cardiovascular, gastrointestinal, and urogenital dysfunctions (Rafanelli et al. 2019), as are observed in Parkinson’s disease (Chen et al. 2020) and other synucleopathies (Mendoza-Velasquez et al. 2019) or immune-mediated ailments, such as multiple sclerosis (Pinter et al. 2015; Ernsberger 2019).

Homeostatic control is communicated by peripheral autonomic neurons in the parasympathetic and sympathetic nervous system (Langley 1921; Jänig 2006), which provide target-specific neuronal pathways that act on a variety of target organs, especially those associated with the cardiovascular system and pelvic organs, but also skin, bones, fat, lymphatic tissue, and the various internal organs. These two domains of the ANS are complemented by a third domain, the enteric nervous system, which was distinguished in the early definition of the autonomic nervous system (Langley 1921) and is composed of two intramural nerve plexuses that traverse and innervate the gut, regulating its activity. In the pelvic region, another nerve plexus, including extramural and intramural ganglia, innervates terminal gut segments and the urogenital system, to orchestrate their activities. The developmental history and nomenclature associated with the ANS have received critical attention in recent years (Espinosa-Medina et al. 2016; Ernsberger and Rohrer 2018; Horn 2018). Here, we chose to discuss the pelvic ganglia and plexus, in comparison with the cranial parasympathetic system and the cervical and trunk sympathetic ganglia and neurons. The distinct cellular and histological organization of the neuronal cell clusters arranged along the autonomic pelvic nerve fiber tracts, together with their mixed neurotransmitter and modulator constellations, which only partially overlap with either sympathetic or parasympathetic neurochemistry, render this choice obvious. The enteric nervous system is similarly distinguished from the pelvic plexus (PP) by distinct histological, neurochemical, and developmental organizations but will not be discussed here.

With the discovery of acetylcholine (Dale and Feldberg 1934) and noradrenaline (von Euler 1966), which are the key transmitters of the sympathetic and parasympathetic systems, respectively, the highly fruitful study of ANS physiology and biochemistry began. The initial characterization of these two classical neurotransmitters was further extended by the recognition of a range of neuromodulators, including purines, nitrogen monoxide (NO), and neuropeptides, which are involved in autonomic signaling processes (Lundberg 1996; Burnstock 2013; Ignarro 2019).

With the characterization of the enzymes involved in the synthesis of neurotransmitters and modulators and the advent of immunohistochemistry, RNA in situ hybridization, and single-cell RNA sequencing, the cellular and molecular characterization of peripheral neuronal elements and their development became possible. The results of these studies, performed in three rodent species and humans, will be discussed in this review. The expression of the rate-limiting enzyme required for catecholamine (CA) biosynthesis, tyrosine hydroxylase (TH), and the enzyme dopamine beta-hydroxylase (DBH), which is located within synaptic vesicles and completes the noradrenaline biosynthesis cascade, or the identification of their transcripts are considered to be prerequisite histological evidence for a noradrenergic transmitter phenotype (Ernsberger and Rohrer 1996). The presence of transcripts for the vesicular monoamine transporter type 2 is used as a marker during RNA sequencing but is less frequently referenced during histochemical analyses (Ernsberger et al. 2017). To define the cholinergic transmitter phenotype, transcripts and enzyme immunoreactivity associated with the synthesizing enzyme choline acetyltransferase (ChAT) or the vesicular acetylcholine transporter (VAChT) are both regarded as crucial markers (Weihe et al. 1998; Ernsberger and Rohrer 1999). To characterize the nitrergic transmitter phenotype, immunoreactivity or transcripts for the neuronal isoform of NO synthase (NOS) is considered to represent strong evidence of NO-producing metabolic synthesis capacity (Beesley 1995). A marker used earlier was NADPH activity. These marker systems are considered to represent valid indicators of the synthesis and vesicular storage (in the cases of noradrenaline and acetylcholine) or activity-regulated synthesis (in the case of NO) of the indicated neurotransmitters. Thus, the terms noradrenergic, cholinergic, and nitrergic in this review will refer to the histological demonstration that the analyzed neurons can synthesize and release the respective signaling molecules.

### A brief overview of small-molecule neurotransmitter distribution

An instructive panorama of the divergent histological and neurochemical organization principles associated with different neuron types first became apparent with the visualization of CA using the histofluorescence technique (Falck et al. 1962). Using this technique, the largely CA character of the superior cervical ganglion (SCG) in rats could be quantitatively analyzed (Yamauchi and Lever 1971). Moreover, the developmental profile associated with CA properties in sympathetic neurons could be studied (Cochard et al. 1978). In contrast to the sympathetic ganglia, which consist of densely packed CA neurons, the principal neurons in the cranial autonomic ganglia are devoid of CA histofluorescence, as was observed in the adult rat ciliary ganglion (CIL) (Landis et al. 1987) and the otic (OG) and sphenopalatine ganglia (SPG) (Leblanc et al. 1987; Leblanc and Landis 1989). The neurons in these ganglia express cholinergic properties. Unlike the cervical and trunk sympathetic ganglia and the cranial parasympathetic ganglia, the rat pelvic ganglia are composed of groups of both CA-positive and -negative cell clusters (Dail et al. 1975). Their composition as a mixture of noradrenergic, cholinergic, and nitrergic structures, will be discussed below.

With the availability of antibodies against transmitter-synthesizing enzymes and neuropeptides, the general understanding of autonomic neurochemistry diversity became greatly advanced. An informative example for several studies in this field is the work by Ceccatelli et al. (1994), who performed a semiquantitative analysis of the detection of a neurotransmitter synthesizing enzyme and neuropeptide immunoreactivity (IR), in several parasympathetic cranial ganglia and major ganglia of the sympathetic trunk, which uncovered major differences in neurotransmitter expression between the parasympathetic and the sympathetic ANS branches.

### The organization of the autonomic ganglia and plexus along the body axis

The cellular resolution provided by immunohistochemistry protocols has advanced the current understanding of the cellular components that comprise the autonomic ganglia and underline the diverse distributions of noradrenergic, compared with cholinergic, and nitrergic neurons, across the autonomic ganglia. The dramatic alteration in neurotransmitter and modulator expression occurs together with changes in the cellular organization from the cranial parasympathetic to the paravertebral and prevertebral sympathetic ganglia, and finally to the pelvic ganglia and plexus.

The cranial ganglia of the parasympathetic nervous system appear as four paired ganglionic structures that are associated with the cranial nerves: the ciliary (CIL), sphenopalatine (SPG) (in humans pterygopalatine, PTG), otic (OG), and submandibular ganglia (SMG) (Gaskell 1886; Fritzsch et al. 2017). An exception pose the dispersed cell clusters of the choroideal ganglion cells (May et al. 2004). The neurons of all cranial ganglia are generated from Schwann cell precursor-like progenitors, distinct from trunk and pelvic autonomic neurons (Dyachuk et al. 2014; Espinosa-Medina et al. 2014; Espinosa-Medina et al. 2016).

The paravertebral sympathetic ganglia are integrated into a macroscopically prominent structure, the sympathetic chain, which was initially considered to be a nerve and was named the intercostal nerve (Winslow 1732). This structure is composed of segmentally arranged pairs of ganglia, connected by commissural strands that are composed of pre- and post-ganglionic sympathetic nerve fibers. The cranial end of the sympathetic chain, with the superior cervical ganglion (SCG) shows a certain degree of lobulation. In the neck region, a middle cervical ganglion (MCG) is observed in larger mammals, including humans. The SCG and MCG are connected, via longitudinal fiber trunks, with the thoracic through sacral paravertebral ganglia.

At the level of the celiac, mesenteric, and renal arteries, which branch from the aorta, a set of less well-demarcated and more lobulated ganglia complement the paired sympathetic strands, including the prevertebral celiac, superior, and inferior mesenteric or aorticorenal ganglia belonging to the preaortic ganglia and plexus. In humans, an additional, less discrete ganglionic structure was recently described, referred to as the prehypogastric ganglion (Beveridge et al. 2016; Furlan et al. 2016). These ganglia are connected with the aortic plexus, which extends into the superior hypogastric plexus, containing a yet unspecified number of neuronal cells (Kraima et al. 2015).

The hypogastric nerves form bilaterally out of the superior hypogastric plexus, which connects them with the aortic plexus and the inferior mesenteric ganglia (IMG). These nerves reach the pelvic ganglia or plexus, which appear more or less discrete and circumscribed, depending on the mammalian species examined (Wozniak and Skowronska 1967). The diverse histological organization observed among varying mammalian species appears to be related to the size of the adult animals. In small animals, prominent major anterior pelvic ganglia (APG) develop, linked to the hypogastric nerve, as observed in rats (Dail et al. 1975; Arellano et al. 2019) and guinea pigs (Morris and Gibbins 1987), where they are connected, via fiber tracts, with smaller accessory ganglia. In larger animals, the system is organized as a plexus consisting of large numbers of smaller neuron clusters, linked by axon tracts, as can be observed in dogs (Li and Masuko 2001) and humans (Takenaka et al. 2005).

Overall, the organization of the peripheral ANS along the body axis changes, from relatively well-demarcated but small and partially lobulated ganglia in the cranial region, to large, discrete ganglia in the cervical and paravertebral domain, to a combination of plexuses and macroscopically less well-demarcated ganglia in the abdominal region. The neurochemical fingerprints of the neurons located in these ganglia and plexuses in rats, guinea pigs (when available), and humans will be discussed here. Comparisons between rats and guinea pigs, which are the first mammalian model species that were analyzed by immunohistochemistry, suggested the conservation of gross organizational features between closely related mammalian species. This conservation extends to differences between the cholinergic and nitrergic characters of the cranial parasympathetic ganglia and the largely noradrenergic properties of the paravertebral and prevertebral sympathetic ganglia in the cervical and trunk regions, which reflect the key divide between parasympathetic and sympathetic features that have been resolved in physiological and pharmacological studies.

This comparative anatomical and cell biological approach can help appreciate and identify altered and new elements of the system, which can be defined when comparing between different genders within the same species and when examining the evolutionary differences that exist between rodents and humans. The significant expansion of the noradrenergic neuronal population in the human pelvic plexus (PP), compared with the rodent major pelvic ganglia (MPG), illustrates one such alteration in neuron populations. The appearance of divergent neuropeptide combinations between male and female rodents in the same species supports the existence of sex-specific differences in neuron populations, which have not yet been analyzed in humans. In addition, the developmental data examining the acquisition of these phenotypes, which has been obtained from studies performed in mice, will also be considered. These studies focus on the central questions of how such divergent neuron populations come into existence and how distinct neurochemical profiles become realized in the different rostrocaudal domains of the ANS and are generated from distinct precursor cells.

## Cranial autonomic ganglia: cholinergic and nitrergic neurons, derived from Schwann cell precursor-like progenitors

### Neurochemical features of the principal neurons in the cranial ganglia of the rat

The availability of antisera against TH, NOS, and ChAT has facilitated the characterization of neuron classes in the rat cranial ganglia (Table 1) and provided evidence regarding the putative neurotransmitters used by individual cells. In CIL and OG, virtually all principal neurons display ChAT immunoreactivity, indicating cholinergic metabolism and transmission (Landis et al. 1987; Leblanc et al. 1987); however, VAChT was not examined in these studies. In the SPG, considerable variability of ChAT staining was observed, with 25% of the principal neurons displaying only very weak or no immunoreactivity (Leblanc et al. 1987). However, the majority of neurons in this ganglion (73%) were positive for NOS (Warn et al. 1997), demonstrating the importance of nitrergic metabolism and transmission in these neurons. Similarly, many neurons in the SMG display NOS IR (Ceccatelli et al. 1994).

**Table 1:** The expression of cholinergic, nitrergic, and noradrenergic transmitter synthesis enzymes in rat and human cranial parasympathetic ganglia

Cholinergic		CIL	OG	SPG	PG	
ChAT	Rat	Virt. all				(Landis et al. 1987)
		All	All	< 100%		(Leblanc et al. 1987)
Nitrergic		CIL	OG	SPG	SMG	
NOS	Rat			majority 40%+++ 30%+		(Warn et al. 1997)
				70 to 80%		(Nozaki et al. 2016)
	Human		75 to 80%	75 to 80%		(Uddman et al. 1999)
Noradrenergic		CIL	OG	SPG	SMG	
CA	rat	no	no	no		(Leblanc et al. 1987)
TH		> 30%	< 1%	< 1%		
TH				1.8%		(Leblanc and Landis 1989)
TH		25–40%			No	(Landis et al. 1987)
TH/DBH		3%				
TH	Human	23%				(Kirch et al. 1995)
DBH		no				

The proportion of neurones expressing the cholinergic marker enzyme ChAT, the nitrergic marker enzyme NOS, and the noradrenergic marker enzymes TH and DBH as detected by immunohistochemistry in rat and human cranial ganglia are provided

CIL, ciliary ganglion; OG, otic ganglion; SPG, sphenopalatine ganglion; SMG, submandibular ganglion; TH/DBH, percentage of TH and DBH double-positive cells

In addition to these small-molecule neurotransmitter phenotypes, the neuropeptides neuropeptide Y (NPY) and vasoactive intestinal peptide (VIP) constitute important markers for cranial neurons. NPY is expressed in 60 to 80% of OG cells, 30% of CIL cells, and 15% of SPG cells in adult rats (Leblanc et al. 1987; Kuwayama et al. 1988). Virtually all NPY-positive cells in the OG and the SPG also express VIP (Leblanc et al. 1987). Many NOS-positive cells in the SPG and SMG also present NPY and/or VIP IR (Ceccatelli et al. 1994) (compare, however, Csati et al. 2012); however, some NPY- and VIP-positive cells are NOS-negative. NPY and TH expression in the SPG appear to occur in different cells (Kuwayama et al. 1988), whereas the majority of NPY IR cells in the CIL are TH-positive, and many are also ChAT-positive (Leblanc et al. 1987).

Unexpectedly, a variable number of TH IR cells have been observed, with none appearing in the SMG, low numbers in the OG and SPG, and high numbers in the CIL (25 to 40% of total cells) (Landis et al. 1987; Leblanc et al. 1987) (Table 1). Importantly, only 3% of cells were identified as TH/DBH double-positive, indicating that in the cranial parasympathetic neurons of rats, the coordinated induction of the NA biosynthetic pathway only occurs in a small number of cells.

Thus, ChAT and NOS constitute the key small-molecule neurotransmitter markers in rat cranial parasympathetic ganglia, whereas the co-expression of noradrenaline-synthesizing enzymes is rare. The percentages of NPY- and VIP-positive neurons differ strongly among ganglia, indicating that they may be associated with target fields.

### The prevalence of NOS-positive cells and the lack of TH/DBH coexpression observed in human cranial parasympathetic ganglia are comparable with observations in rat

In the human ciliary ganglia, approximately 23% of neurons were TH IR-positive, based on examinations of adult body donors (Kirch et al. 1995). Similar to observations made in the rat cranial parasympathetic ganglia, and different from the observations in the rat and human superior cervical ganglion, these neurons did not co-express DBH (Table 1), indicating that they are unable to synthesize noradrenaline. TH-positive cells can be detected during fetal development, between the 12th and 15th post-conception weeks, at low levels, in the region of the ciliary and submandibular ganglion (Kiyokawa et al. 2012; Teshima et al. 2019).

At this early developmental stage, strong NOS IR can be detected in the developing submandibular gland, in the area of the developing SMG, whereas the signals in the area of the SPG and OG were weak to moderate, and the signals in the developing domain of the CIL were weak or absent (Kiyokawa et al. 2012). In the SPG and OG of adult donors, 75 to 80% of neurons were NOS-positive (Uddman et al. 1999).

VIP IR has also been described during the 12th to 15th fetal weeks in the SPG, SMG, and CIL, at different intensity levels and with different patterns, with only a few VIP-positive cells observed in the region of developing CIL (Kiyokawa et al. 2012). In adult donors, more than 90% of cells were VIP-positive in the SPG and OG (Motosugi 1993; Uddman et al. 1999), and VIP and NOS IR were reported to colocalize in the SPG (Csati et al. 2012). In stark difference to the observations for the SPG, no VIP-positive neurons were described in the CIL of adult donors (Kirch et al. 1995). Among choroid ganglion cells in young and adult donors, 95% were VIP-positive (May et al. 2004). No NPY-positive cells were detected in the CIL of elderly donors (Kirch et al. 1995). NPY-positive choroid ganglion cells represented fewer than 5% of total cells, compared with 95% that were VIP-positive (May et al. 2004).

Similar to the situation observed in rats, the co-expression of noradrenaline-synthesizing enzymes was not observed in human cranial parasympathetic ganglia, even though TH could be detected. Although the systematic analysis of cholinergic markers by IHC is not currently available, the detection of NOS shows similarities between humans and rats (Table 1). The limited data on neuropeptide IR in the cranial ganglia indicate possible differences between species, with NPY being more abundant in rats than humans.

### Embryonic development of the mouse cranial parasympathetic ganglia

In two milestone studies (Dyachuk et al. 2014; Espinosa-Medina et al. 2014), the development of the parasympathetic ganglia was shown to depend on cranial nerves and neural crest-derived Schwann cell precursors, which are dispersed along these nerves. These cells are initially derived from Sox10-positive neural crest cells, which become Sox10/Phox2b-double positive as the nerve grows. They also express characteristic neural crest cell markers, such as FoxD3 or Sox2, and Schwann cell precursors makers, like ErbB3 and PLP (Dyachuk et al. 2014; Espinosa-Medina et al. 2014).

The proneural gene Ascl1 must be upregulated to achieve neuronal differentiation. The mutational inactivation of Ascl1 results in the severe atrophy or lack of newborn cranial parasympathetic neurons (Hirsch et al. 1998). Phox2 transcription factors (TFs) are also necessary. In newborn Phox2a-mutant animals, the OG and SPG are missing, whereas the SMG is only partially lost at birth (Morin et al. 1997). These observations demonstrated the specific effects on the rostral parasympathetic ganglia. Together with Phox2b, which is necessary for the development of both parasympathetic and sympathetic postganglionic neurons (Pattyn et al. 1999), Phox2a and Ascl1 are expressed in both parasympathetic and sympathetic neurons (see below).

The expression of Hand2, a TF that is expressed in sympathetic neurons and is required for noradrenergic differentiation (Lucas et al. 2006; Morikawa et al. 2007; Schmidt et al. 2009), has also been documented in embryonic cranial parasympathetic neurons. Hand2-positive neurons in the developing SPG (Stanzel et al. 2016) and SMG (Teshima et al. 2019) are found in regions where DBH or TH can be detected. However, the mutational inactivation of Hand2 in already-differentiated cells does not affect DBH expression, which is transient and disappears during the advanced embryonic stages (Stanzel et al. 2016). This lack of effect on DBH expression in the parasympathetic ganglia is not understood mechanistically, but aligns with the lack of TH and DBH co-expression in these neurons.

Hmx2 and 3 are selectively expressed in mouse cranial parasympathetic ganglia, during embryonic development (Espinosa-Medina et al. 2016), whereas Hmx1 is expressed in sympathetic ganglia (Furlan et al. 2013). In addition, sympathetic neurons differ from cranial parasympathetic neurons by the expression of Hand1 and Gata3. While roles for the latter two TFs in the development of sympathetic neurones have been documented as described below, the importance of HMX2 and 3 in parasympathetic neurodevelopment and expression of the transmitter-synthesizing enzymes or neurotransmitter phenotype is not resolved.

### Cranial parasympathetic neuron summary

Taken together, the data obtained from humans, rats, and mice demonstrate a relatively complex neurotransmitter and peptide expression pattern, with the cholinergic and nitrergic transmitter synthesizing enzymes ChAT and NOS, respectively, expressed in most if not all neurons in the parasympathetic cranial ganglia. The noradrenergic transmitter synthesizing enzymes TH and DBH can be expressed, transiently, but do not appear to be co-expressed in a significant number of neurons. Correspondingly, cranial parasympathetic ganglion neurons do not show CA histofluorescence, indicating that no substantial loading of synaptic vesicles with CA occurs, and cranial parasympathetic neurons do not secrete noradrenaline as a neurotransmitter.

Cranial parasympathetic postganglionic neurons in mice are generated from Schwann cell precursor-like progenitor cells, which distribute along the nerves to reach their final destinations. Starting as Sox10-positive neural crest phenotypes, the cells progress through a Schwann cell precursor-like Sox-10/Phox2b double-positive state to an Ascl1-positive state. Although these TFs are also expressed during the development of sympathetic postganglionic neurons, the parasympathetic postganglionic lineage does not exhibit the coordinated expression of noradrenergic biosynthesis enzymes. How TFs that are uniquely expressed in parasympathetic progenitor cells, such as Hmx2 and 3, contribute to the transmitter phenotype requires further investigation.

## Paravertebral and prevertebral sympathetic ganglia: largely noradrenergic neurons, derived from migrating neural crest precursors

### Transmitter-synthesizing enzyme and neuropeptide expression patterns in human cervical and stellate sympathetic ganglia

In the superior cervical ganglia of adult human donor tissues, virtually all neurons were described as both TH- and DBH-positive (Baffi et al. 1992; Kirch et al. 1995) (Table 2). These studies confirmed that in humans, the vast majority of SCG neurons are noradrenergic. Later studies examining TH IR cell numbers yielded proportions below 90% (Tajti et al. 1999; Kokubun et al. 2019). In a detailed IHC analysis of TH and DBH expression in the superior (SCG), middle cervical (MCG), and stellate ganglia (STG), DBH was observed in more than 90% of neurons (Kokubun et al. 2019).

**Table 2:** The expression of noradrenergic markers in adult human sympathetic ganglia

	SCG	MCG	STG	Thoracic	Sacral	
TH	83.8%	59.3%	70.4%			(Kokubun et al. 2019)
				75%		(Schalling et al. 1989)
	> 75%					(Tajti et al. 1999)
					90%	(Takenaka et al. 2005)
	Virt. all					(Kirch et al. 1995)
	Rich					(Baffi et al. 1992)
DBH	91%	92.1%	94.2%			(Kokubun et al. 2019)
				75%		(Schalling et al. 1989)
	Virt. all					(Kirch et al. 1995)
	Similar to TH					(Baffi et al. 1992)

The proportion of neurons immunopositive for the noradrenergic marker enzymes TH and DBH for different human cervical and paravertebral sympathetic ganglia are provided

SCG, superior cervical ganglion; MCG, middle cervical ganglion; STG, stellate ganglion. Virt. all indicates that virtually all neurons are positive; rich indicates a rich supply of positive neurons; similar TH indicates that the number of DBH and TH-positive cells are similar as expected for cells co-expressing the noradrenergic marker enzymes

The evaluation of T2 and T3 sympathetic ganglia showed that 75% of neurons were TH- and DBH-positive (Schalling et al. 1989), and in the sacral sympathetic ganglia, approximately 90% of neurons were TH-positive (Takenaka et al. 2005). This latter study was particularly interesting as it compared the percentages of TH-positive cells in more caudoventrally located autonomic neurons. The percentage of TH-positive neurons changes, from approximately 90% in the sacral sympathetic ganglia to 58% along the hypogastric nerve, 36% in the pelvic splanchnic nerves, 46% in pelvic neurovascular bundles, and 58% in the pelvic plexuses with highly variable counts among individuals. This study nicely illustrates the sharp loss of noradrenergic neuron predominance moving toward the caudal and ventral positions along the hypogastric nerves.

Systematic studies examining the expression of cholinergic markers, such as ChAT and VAChT, and the nitrergic marker NOS are not available in humans. Only a single report exists describing the lack of NOS IR neurons in the SCG (Tajti et al. 1999), indicating that nitrergic signaling does not play a significant role in transmission from sympathetic neurons located in the paravertebral sympathetic ganglia, which is similar to the pattern observed in rodents.

Quantitative data analyzing VIP in the human SCG have provided varying results (Tajti et al. 1999; Kokubun et al. 2019). Using sensitive 3,3′-diaminobenzidine (DAB) IHC, 16 and 19% of neurons were found to be VIP-positive in the SCG and MCG of adult donors, respectively (Kokubun et al. 2019). The co-localization of VIP and calcitonin gene-related peptide (CGRP) has been reported in the SCG of donors of various ages (Schmitt et al. 1988; Baffi et al. 1992). VIP and CGRP are also co-localized in the human STG, which shows a similar percentage of VIP IR cells (14%) (Schmitt et al. 1988; Kokubun et al. 2019). Developmental analysis has been performed for VIP in the human STG, which demonstrated a strong decrease in the proportion of VIP-positive cells from the neonatal to the adult stages (Roudenok 2000). In the lumbar paravertebral sympathetic ganglia, some VIP-positive cells are detected, all of which were TH-negative (Jarvi et al. 1989), suggesting a cholinergic phenotype.

NPY has been detected in a large number of human SCG neurons, most of which are TH-positive (Baffi et al. 1992; Kirch et al. 1995). Quantification revealed that 75% of neurons in the SCG were TH- and NPY-positive (Tajti et al. 1999), 59% and 71% NPY-positive cells in SCG and MCG, respectively and 87% in STG (Kokubun et al. 2019). One half of the TH- and DBH-positive cells in T2 and T3 sympathetic neurons have been described as NPY-positive (Schalling et al. 1989). During development, NPY-positive neurons represented 7% of the paravertebral ganglia in premature fetuses, from 24 to 27 weeks, and the proportion increased to 41% by 38 to 41 weeks of gestation (Roudenok 2000).

The studies have shown that human sympathetic ganglia are composed of TH- and DBH-positive noradrenergic neurons, which largely co-express the neuropeptide NPY. VIP expression was found in a small neuron population in the superior and middle cervical ganglia and at a slightly higher percentage in the STG. IHC data examining cholinergic and nitrergic transmitter synthesizing enzymes could not be retrieved from the major online literature databases. Similarly, systematic studies examining the prevertebral sympathetic ganglia were not available. However, these aspects have been studied, in detail, in several rodent species.

### The predominance of noradrenergic properties, in combination with changing neuropeptide expression patterns, in the guinea pig para- and prevertebral ganglia

A systematic comparison of the two key enzymes associated with the noradrenaline biosynthesis cascade, TH and DBH, has been performed in guinea pigs, using IHC-based methods. These studies demonstrated that the vast majority of neurons in the SCG (Lundberg et al. 1982), the thoracolumbar paravertebral ganglia (Gibbins 1992), and the celiac ganglion/superior mesenteric ganglionic (CEG/SMG) complex (Hokfelt et al. 1977; Lundberg et al. 1982; Lindh et al. 1986) expressed both TH and DBH. A DBH IHC analysis is not available for the IMG. However, more than 95% of neurons showed TH IR (Sann et al. 1995; Parr and Sharkey 1996), and CA histofluorescence analysis demonstrated very few nonfluorescent cells among the vast majority of densely packed fluorescent cells (Furness and Costa 1973). Taken together, these studies indicated the existence of a predominant noradrenergic transmitter phenotype along the entire rostrocaudal axis of the sympathetic trunk.

This vast dominance of noradrenergic neurons throughout the paravertebral and prevertebral sympathetic ganglia changes only along the hypogastric nerve, where the neurochemistry of ganglion-like neuron clusters shifts to a mixed fluorescent and nonfluorescent character (Furness and Costa 1973). This feature continues to be retained in the anterior and posterior pelvic plexuses of autonomic cells. Similarly abrupt changes are also observed for cholinergic and nitrergic transmitter phenotypes, with fewer than 5% of neurons identified as ChAT IR in the IMG (Elfvin et al. 1993; Sann et al. 1995) and the detection of only occasional NOS-positive cells (Elfvin et al. 1993; Parr and Sharkey 1996), in contrast with the high proportion of ChAT- and NOS-positive cells in the pelvic ganglia (see below)

A detailed study examining the small population of ChAT-expressing neurons was performed in the IMG (Sann et al. 1995). Although 95% of the IMG neurons were TH-positive, slightly less than 5% of these neurons were ChAT-positive, with a high degree of mutual exclusion: 94% of TH-negative cells were ChAT-positive, indicating that almost all neurons can be grouped into either noradrenergic or cholinergic subpopulations. The cholinergic neurons were significantly larger than the noradrenergic neurons, and the majority of ChAT-positive cells (64%) appeared to be clustered in the caudal lobe, near the hypogastric nerve. The observation that only occasional ganglionic neurons were NOS-positive complements this observation (Elfvin et al. 1993; Sann et al. 1995; Parr and Sharkey 1996). These NOS IR neurons, which comprised 0.9% of the ganglion neurons, were TH- and ChAT-negative, with smaller cell body sizes than the other cell populations (Sann et al. 1995). Thus, the IMG neurons can be classified into three largely distinct groups: a vast majority of noradrenergic cells, a minor population of cholinergic cells, and a minuscule population of nitrergic neurons, which can be distinguished by neurochemistry and cell body sizes.

Using triple-labeling immunohistofluorescence, the characterization of post-ganglionic sympathetic neuron subpopulations was refined, to analyze the expression patterns of neuropeptides and the synthesizing enzymes required for small-molecule neurotransmitters (Gibbins 1992). In addition, the neurochemical features of the synaptic baskets of innervating pre-ganglionic neurons were explored. By combination with retrograde labeling from target tissues, neurochemical landmarks for certain target-selective pathways could also be defined. This type of analysis revealed three populations of post-ganglionic neurons in the lumbar sympathetic ganglia of the guinea pig and their putative targets. Staining for TH and NPY distinguished TH- and NPY-positive neurons from TH-positive noradrenergic neurons devoid of NPY. In addition, TH-negative and VIP-positive neurons that co-express NPY and are presumably cholinergic were also detected (Gibbins 1992). The exclusive association of substance P-positive synaptic baskets with VIP^+^/NPY^+^ cells and their retrograde labeling from the muscle provided evidence that these neurons can be classified as skeletal muscle vasodilators. The CGRP-positive staining of synaptic baskets surrounding TH^+^/NPY^+^ cells that were retrogradely labeled from the skeletal muscle indicated that this population of neurons functions as vasoconstrictors. Finally, the TH^+^/NPY^−^ neurons that were retrogradely labeled from hairy skin were regarded as pilomotors (Gibbins 1992).

Although 46 and 60% of the principal neurons in the thoracic and lumbar sympathetic ganglia, respectively, are TH and NPY double-positive, only 11 and 13%, respectively, were double-positive for VIP and NPY but lacked TH (Gibbins 1992). In the celiac ganglion, a population of small TH^–^/VIP^+^/NPY^+^ neurons has also been described (Lindh et al. 1986), which also appear to lack DBH expression (Lundberg et al. 1982). The size of this population is much smaller than in the paravertebral ganglia, however, and this population represents less than 1% of VIP-positive cells. Interestingly, VIP-positive neurons encompass a small number of larger-sized neurons that express TH, NPY, and VIP (Lindh et al. 1986). This observation indicates diversity, even within the small population of VIP-positive neurons, which is further confirmed by the observation that only 0.2% of the VIP-positive neurons in the lumbar ganglia are NOS IR (Morris et al. 1998).

The expression patterns of the neuropeptides NPY and somatostatin (SOM), which are expressed in noradrenergic sympathetic neurons in the CEG/SMG, appear to be largely but not completely mutually exclusive (Lundberg et al. 1982; Lindh et al. 1986). Similar to the situation observed in the thoracolumbar sympathetic ganglia, NPY expression in prevertebral ganglia is not restricted to noradrenergic neurons but can also be detected in the vast majority of the small population of ChAT-positive neurons, as analyzed in the IMG (Sann et al. 1995). Interestingly, the relative abundance of NPY- and SOM-positive cells in the CEG/SMG appears to be opposite that in the IMG (Table 3). Although two thirds of the neurons in the CEG/SMG express NPY (Lindh et al. 1986; Sann et al. 1995), only 20 to 25% of neurons were NPY-positive in the IMG (Sann et al. 1995; Parr and Sharkey 1996). An inverse pattern was observed for SOM expression. Together with the uneven distribution of SOM within the CEG/SMG (Hokfelt et al. 1977), these data showed the changing proportions of neuropeptide-positive cells across the sympathetic ganglia and indicated an important association between neuropeptide expression, neuronal position, and target innervation. However, the importance of the neuron-target interaction for the establishment of neuropeptide expression was only demonstrated for the neuropeptide VIP, in rat and mouse cholinergic sympathetic sudomotor neurons, and for cortistatin expression, in parasympathetic ciliary neurons (Darland et al. 1995; Darland and Nishi 1998; Nishi et al. 2009).

**Table 3:** Neuropeptide expression in sympathetic ganglia of adult guinea pigs

	SCG	Thoracic	Lumbar	CEG/SMG	IMG	
VIP		11%	13%			(Gibbins 1992)
				< 1%		(Lindh et al. 1986)
					1 to 2%	(Parr and Sharkey 1996)
NPY		57%	73%			(Gibbins 1992)
				65%		(Lindh et al. 1986)
					App. 20%	(Parr and Sharkey 1996)
					App. 22%	(Sann et al. 1995)
SOM	Single			Significant		(Lundberg et al. 1982)
				25%		(Lindh et al. 1986)
	Single			59% in AI, 25% in PS	62.5%	(Hokfelt et al. 1977)
					App. 80%	(Sann et al. 1995)
					App. 80%	(Parr and Sharkey 1996)

The proportion of neurones immunopositive for the neuropeptides VIP, NPY and SOM in different sympathetic ganglia are provided

SCG, superior cervical ganglion; CEG/SMG, celiac ganglion/superior mesenteric ganglia; IMG, inferior mesenteric ganglion; AI, anterior–inferior part of the ganglion complex; PS, posterior-superior part of the ganglion complex; “single” and “significant” refer to only single cells as compared with significant numbers of cells in the ganglion. “App.” indicates approximate estimates

### The distribution and developmental expression of biosynthetic enzymes for classical neurotransmitters and neuropeptides in the rat sympathetic ganglia

Similar to the pattern observed in the guinea pig, approximately 90% of neurons in the rat superior cervical ganglion, stellate ganglion, and celiac ganglion express TH (Masliukov and Timmermans 2004; Maslyukov et al. 2010). Although only occasional ChAT-positive cells are present in the SCG (Maslyukov et al. 2010), CEG (Maslyukov et al. 2010), and IMG (Sann et al. 1995), a significant number (up to 5%) of ChAT-positive neurons can be detected postnatally in the STG (Morales et al. 1995; Masliukov and Timmermans 2004). TH and ChAT co-expression was either not detected (Morales et al. 1995) or detected only in a very small percentage (less than 1%) of cells (Masliukov and Timmermans 2004). Single NOS-positive neurons in the SCG were only reported by one group (Ceccatelli et al. 1994), but not by others (Alm et al. 1995; Masliukov et al. 2014), who also could not detect them in the STG and CEG.

Particular attention was paid to the expression of the neuropeptides VIP and NPY (Table 4). In postnatal rats, VIP was detected only occasionally, in the SCG (Masliukov and Timmermans 2004) and the CEG/SMG (Maslyukov et al. 2010), whereas in the STG, virtually all ChAT-positive neurons co-expressed VIP (Morales et al. 1995). The developmental time course of VIP and ChAT expression in the STG and the number of VIP-positive neurons are closely associated (Masliukov and Timmermans 2004). Some VIP-positive STG neurons also co-express CGRP and SOM, but not NPY (Morales et al. 1995; Masliukov and Timmermans 2004; Masliukov et al. 2012). A small number of VIP-positive cells that co-express TH has been described (Masliukov and Timmermans 2004), demonstrating that the expression of VIP is not entirely restricted to cholinergic cells. VIP-positive cell bodies in the STG are not NOS-positive, however (Ceccatelli et al. 1994). Single-cell RNA sequencing data from the mouse thoracic sympathetic ganglia, however, disclosed the virtual absence of VIP (as well as SOM and CGRP) from noradrenergic neurons, compared with NPY, which is not strictly correlated with any particular small-molecule neurotransmitter phenotype (Furlan et al. 2016); see table 1 in Ernsberger and Rohrer (2018).

**Table 4:** Development of VIP and NPY expression in the rat sympathetic ganglia

	Onset	% at onset	P0	P10	P60	
VIP						
SCG	E14.5	App. 30%	2%			(Tyrrell and Landis 1994)
STG			5%	6%	3%	(Masliukov and Timmermans 2004)
NPY						
SCG, STG	E12.5	Almost all	55%			(Tyrrell and Landis 1994)
SCG			50%	57%	65%	(Masliukov et al. 2012)
STG			40%	45%	55%	(Masliukov and Timmermans 2004)
CEG			65%	62%	80%	(Masliukov et al. 2012)

The proportions of neurons that are immunopositive for the neuropeptides VIP and NPY in rat sympathetic ganglia at different developmental stages are provided. The expression onset is provided with the embryonic day (E) of first marker detection and the proportion (%) of positive cells on the day of expression onset. The proportion of positive cells is also provided for postnatal (P) day 0 to 60 animals

SCG, superior cervical ganglion; SCG, stellate ganglion; CEG, celiac ganglion

During the development of the SCG but not the STG, VIP IR, at different signal intensities, has been detected, starting at E14.5, in a subset of TH-positive cells (Tyrrell and Landis 1994). At this stage, VIP IR was detected in one third of the ganglion cells, which reduces to only 2% at birth. In both the rat SCG and STG, NPY IR was first detected at E 12.5, at similar signal intensities in almost all TH-positive cells (Tyrrell and Landis 1994). At birth, approximately 55% of cells in both ganglia remained NPY-positive. The immunohistochemical results were confirmed by in situ hybridization for the respective mRNAs, demonstrating the occurrence of early induction during embryonic development, followed by the subsequent restriction to specific subpopulations. Interestingly, both peptides can be detected in bromodeoxyuridine (BrdU)-labeled precursors and neurons (Tyrrell and Landis 1994).

The NPY-positive neurons eventually constitute the largest subpopulation of noradrenergic neurons in the SCG, STG, and CEG (Jarvi et al. 1986; Tyrrell and Landis 1994; Hall and MacPhedran 1995; Masliukov and Timmermans 2004; Maslyukov et al. 2010; Masliukov et al. 2012). The vast majority of NPY-positive neurons co-express TH, whereas ChAT-positive cells are generally NPY-negative (Masliukov et al. 2012). In the STG, only a few NPY-positive neurons lacked TH expression, and VIP-positive cells were not found to express NPY (Masliukov and Timmermans 2004).

The development of the full complement of VIP-positive neurons in the rat sympathetic ganglia involves the postnatal, target-dependent induction of cholinergic neurotransmitter properties, including VIP expression in the sympathetic neurons that innervate sweat glands (Landis 1988). In a series of studies, which belong to the founder tales of developmental neurobiology, catecholaminergic sympathetic fibers were found to innervate sweat glands in the rat footpad during early postnatal development and transdifferentiate, losing CA histofluorescence and acquiring cholinergic properties (Landis and Keefe 1983). The cholinergic differentiation process involves the induction of ChAT (Leblanc and Landis 1986) and VAChT (Guidry and Landis 1998). The expression of the neuropeptides VIP and CGRP complements the cholinergic phenotype (Landis and Fredieu 1986). The shift in the transmitter phenotype could be induced by the transplantation of footpad tissues into regions that are normally innervated by noradrenergic sympathetic neurons (Schotzinger and Landis 1988, 1990), as described by key studies on the “target determination of neurotransmitter phenotype in sympathetic neurons” (Schotzinger et al. 1994). The blockade of the corresponding differentiation process, in vitro, through the interference of neuropoietic cytokine signaling in neuron/target co-cultures, indicated that member(s) of this family of cytokines could induce target-dependent cholinergic differentiation (Habecker et al. 1997). Using a conditional knockout of the gp130 cytokine receptor in mouse sympathetic neurons, the essential role played by neuropoietic cytokines during this process was demonstrated in vivo (Stanke et al. 2006), but which of the cytokines expressed in sweat glands is important remains unclear. Eventually, data from human genetic disorders identified cytokine receptor-like factor 1 (CRLF1)/cardiotrophin-like cytokine factor 1 (CLCF1) as cholinergic differentiation factor in human sweat glands (Di Leo et al. 2010; Melone et al. 2014).

### The generation of sympathetic post-ganglionic neurons from migrating neural crest cells: insights from chick and mouse studies

#### Dorsoventral migration of thoracolumbar neural crest cells and the induction of differentiation by aorta-derived bone morphogenetic proteins

Unlike the spread of neural crest-derived precursors along previously formed nerves, as was observed in the head during the formation of parasympathetic ganglia, progenitor cells located at the dorsal crest of the forming neural tube (Kalcheim 2018), which are recruited for the formation of sympathetic ganglia, initially undergo an epithelial-to-mesenchymal transition and migrate ventrally through developing somites (Bronner-Fraser 1995; Young et al. 2004; Kulesa et al. 2009; Kalcheim 2015). Upon arrival in the vicinity of the aorta, the neural crest-derived precursors begin to differentiate and express TH and DBH, as detailed in the chick embryo (Ernsberger et al. 1995; Ernsberger 2000), indicative of their acquisition of a noradrenergic phenotype. Under the influence of bone morphogenetic protein (BMP) 4 and 7, which are expressed in the walls of the aorta, this differentiation process is triggered concurrently with the acquisition of neuronal properties (Reissmann et al. 1996; Shah et al. 1996; Schneider et al. 1999; Patzke et al. 2001). During this event, progenitor and neuroblast cells progress through a sequence of transcription factor induction steps, as shown in chick embryos (Ernsberger et al. 1995; Howard et al. 2000; Tsarovina et al. 2004).

In chick and mouse embryos, cholinergic features can be detected soon after noradrenergic differentiation, and the co-expression of both sets of markers can be detected in a significant number of cells (Ernsberger et al. 1997; Huber and Ernsberger 2006; Furlan et al. 2013; Huang et al. 2013). Thus, developing cells are considered to be hybrid cells during these initial stages (Apostolova and Dechant 2009; Furlan et al. 2013). Subsequently, these neurons with mixed transmitter phenotypes start to segregate into noradrenergic and cholinergic neurons. The regulatory processes that underlie the segregation of these two neurotransmitter phenotypes are not yet fully understood.

#### Induction of a transcription factor network for the generation of the noradrenergic transmitter phenotype

The BMP-induced differentiation, from Sox10-positive neural crest precursors to sympathetic neuroblasts, is critically dependent on the TF Phox2b (Pattyn et al. 1999). Phox2b is not only required to initiate differentiation but is also necessary to maintain the expression of noradrenergic characteristics, in particular TH and DBH expression, in differentiated sympathetic neurons (Coppola et al. 2010). Another critical TF that is involved in the development of the noradrenergic phenotype is Hand2, as demonstrated by the effects of Hand2 mutational inactivation (Lucas et al. 2006; Morikawa et al. 2007). Despite the absence of effects on Gata2/3, Phox2a/b, and Ascl1 expression, Hand2 regulates TH and DBH expression during neuronal differentiation. Importantly, it is required for Hand1 (Morikawa et al. 2005), a TF that is selectively expressed in sympathetic neurons (Espinosa-Medina et al. 2016; Zeisel et al. 2018). In differentiated neurons, Hand2 is required for the expression of the noradrenergic phenotype, as demonstrated in chick sympathetic neurons (Schmidt et al. 2009). Interestingly, in cranial parasympathetic precursors, where its expression appears to be associated with TH expression, Hand2 is not sufficient to drive the co-expression of TH and DBH or initiate full noradrenergic phenotypic differentiation (see above). Gata3 inactivation in mice reduces TH but not DBH mRNA levels in sympathetic ganglia (Tsarovina et al. 2004). Whether the combined activities of Phox2a/b, Hand1/2, Gata2/3, and Ascl1 and their regulation during various developmental phases are sufficient to explain the induction and maintenance of the full set of gene products that are necessary for a *bona fide* noradrenergic transmitter phenotype among sympathetic neurons is currently not fully understood. The role played by Insm2, which is specifically expressed in mature noradrenergic but not cholinergic sympathetic neurons (Table 5), also requires further analysis.

**Table 5:** Expression of selected transcription factors in mouse thoracic sympathetic neurons

	NA1	NA2	NA3	NA4	NA5	Ach1	Ach2
Phox2b	5.3	7.59	5.97	5.89	4.09	5.25	3.38
Phox2a	6.8	5.85	7.61	6.06	5.39	6.12	3.38
Ascl1	0.1	0	0.08	0	0	0	0
Hand1	3.3	4.13	3.48	2.33	3.26	2	1.5
Hand2	7.6	11.87	11.54	11.78	11.87	8	7.75
Gata2	5.8	7.41	6.64	5.72	5.48	2.5	1.38
Gata3	2.8	3.54	3.35	5.06	4.35	3.12	1.88
Hmx1	1.6	1.33	2.17	1.22	1.17	0.25	0
Hmx 2/3	ND	ND	ND	ND	ND	ND	ND
Insm1	0.1	0.05	0.17	0	0.04	0	0
Insm2	1.5	2.05	2.01	2.56	1.48	0	0

The numbers given are mean number of transcripts per cell in the indicated noradrenergic (NA 1–5) and cholinergic (Ach1 and 2) sympathetic neuron subpopulations as analyzed by single cell RNA sequencing (Furlan et al. 2016). Whereas no major differences are observed for Phox2 transcript levels, Hand 1 and 2 transcripts in noradrenergic neurons in general exceed those in cholinergic neurons in number. Similar differences are observed for Gata 2 and 3. Whereas HMX1 is detected at least 5-fold higher levels in noradrenergic than cholinergic neurons, HMX 2 and 3 transcripts are not found in any sympathetic neuron population, neither noradrenergic nor cholinergic. The most striking difference between noradrenergic and cholinergic neurons is observed for Insm2. Data derived from Furlan et al. (2016), supplementary table nn 4376-s4

In noradrenergic neuroblastoma cell lines, core regulatory circuits (CRCs) that govern gene expression programs have been identified by super-enhancer (SE) mapping. The strongest SEs have been observed at loci associated with the TFs Hand2, Phox2b, Phox2a, Gata2, and Gata3, which bind to each other’s SEs and are thought to control noradrenergic identity through cross-regulatory expression (Boeva et al. 2017; van Groningen et al. 2017). The extent to which SE-driven CRCs are active in developing sympathetic neurons remains to be determined.

#### Cholinergic sympathetic neurons can be generated from initially hybrid precursors or, later, from noradrenergic sympathetic neurons

Cholinergic differentiation in sympathetic neurons can be affected by growth factor signaling during both embryonic and postnatal development. During chick embryonic development, the expression of the cholinergic markers ChAT and VIP and the co-expression with the glial cell line-derived neurotrophic factor (GDNF)-family growth factor receptor Ret suggests a role for Ret signaling in the development of cholinergic sympathetic neurons (Ernsberger et al. 1997). During embryogenesis in mice, Ret signaling is required for the normal development of the cholinergic markers ChAT and VAChT and the formation of a normal complement of cholinergic neurons in the STG and upper thoracic sympathetic ganglia (Burau et al. 2004; Furlan et al. 2013).

The early and widespread expression of cholinergic properties in newly formed mouse sympathetic neurons, at E12.5, and their co-expression with noradrenergic properties are rapidly restricted to a small subset of cells during development (Burau et al. 2004; Huber and Ernsberger 2006; Furlan et al. 2013; Huang et al. 2013). The TF homeobox protein 1 (HMX1), which is initially expressed in Ret- and tropomyosin receptor kinase (Trk)C-positive sympathetic precursor cells at E13.5, becomes exclusive to the noradrenergic lineage of sympathetic neurons and is strongly associated with TrkA expression (Furlan et al. 2013). The rapid downregulation of Ret in HMX1-positive neurons results in the restriction of HMX1 to vesicular monoamine transporter (VMAT)2-positive noradrenergic cells and the absence from cholinergic neurons, as early as E14.5. The mutational inactivation of HMX1 results in the massive reduction of TrkA-positive cells in the embryonic sympathetic ganglia and the dramatic reduction of TH expression, without affecting DBH and VMAT2. In addition, the downregulation of Ret, SOM, and VIP is suppressed, whereas ChAT and VAChT are unaffected. HMX1 appears to be required for the expression of one key noradrenergic marker and the segregation of noradrenergic from cholinergic differentiation pathways.

The mutational inactivation of Ret results in the premature (E14.5) increase in HMX1 and TrkA expression and the near-complete absence of ChAT, VAChT, VIP, and SOM expression (Furlan et al. 2013). In addition, a small increase in the precursor marker TrkC is observed. The repressive effects of HMX1 on cholinergic properties are mediated by interference with T-cell homeobox 3 (TLX3) expression. The TF TLX3 is expressed in nearly all precursor cells, at E13.5, and becomes largely mutually exclusive with HMX1 at E15.5 (Furlan et al. 2013). Most TLX3-positive cells are VAChT-positive at E15.5, although some express TH. At P60, TLX3 and TH expression are entirely mutually exclusive. In HMX1 mutant mice, TLX3 fails to become repressed, and TLX3-positive cells retain Ret staining (Furlan et al. 2013). Correspondingly, the mutational inactivation of TLX3, which shows similar distribution patterns as those observed for VAChT and VIP in embryonic and postnatal mice, results in the loss of VIP and SOM expression at early embryonic stages (E12.5), in addition to the loss of VAChT at E18.5 (Huang et al. 2013). In addition, the mutational inactivation of TLX3 resulted in the loss of high-level Ret expression. In contrast, in Ret mutant mice, VIP and SOM expression were unaffected at E12.5 but were abolished at E18.5.

These results demonstrated the critical role played by Ret signaling in the segregation of noradrenergic and cholinergic transmitter properties and the embryonic development of cholinergic sympathetic neurons. In addition, the later upregulation of Ret expression in noradrenergic neurons may play a role during the advanced stages of target innervation in certain tissues (Furlan et al. 2016). The mechanisms underlying this regulation have not yet been resolved.

In contrast with the embryonic regulation of cholinergic differentiation in sympathetic neurons, glycoprotein 130 (gp130) signaling induced by neurokine growth factors is essential for the postnatal induction of cholinergic properties in previously noradrenergic neurons that innervate mammalian sweat glands, as demonstrated by gp130 mutational inactivation in mice (Stanke et al. 2006) and by receptor blockade in cultures of rat sympathetic neurons (Habecker et al. 1997). Several members of the family of neuropoietic cytokines are expressed in developing sweat glands (Stanke et al. 2006). The mutational inactivation of CRLF1 and leukemia inhibitory factor receptor (LIFRβ) in human genetic diseases identified CRLF1/CLCF1 to be essential for cholinergic differentiation of sweat gland innervation (Di Leo et al. 2010; Melone et al. 2014). In contrast, the early, embryonic expression of the cholinergic markers VAChT and the neuropeptide VIP was not affected by interference with the respective receptor subunits LIFR beta or CNTFR alpha interacting with gp130 in mice (Stanke et al. 2000).

#### Summarizing key aspects of sympathetic neuron subpopulations, their development, and comparisons with cranial parasympathetic post-ganglionic neurons

**Fig. 1:**
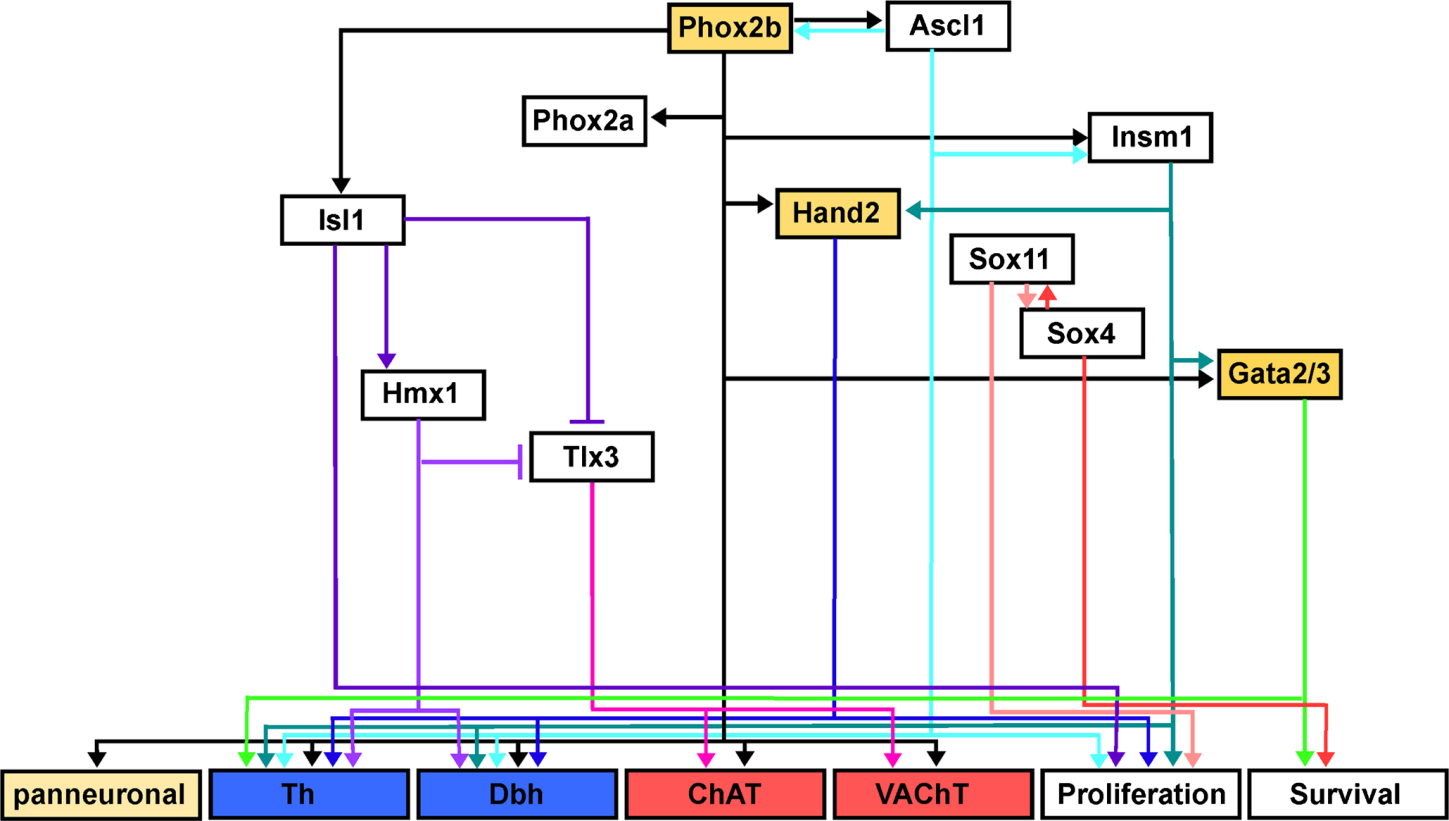
Schematic illustration of the gene regulatory network controlling sympathetic neuron differentiation. Arrows indicate target gene expression, proliferation, or survival affected by transcription factor knockout. Initial development is only affected by Phox2b, Hand2, and Gata2/3. References for individual TFs: Phox2b (Pattyn et al. 1999); Ascl1 (Pattyn et al. 2006); Hand2 (Lucas et al. 2006; Hendershot et al. 2008; Schmidt et al. 2009); Gata2/3 (Lim et al. 2000; Tsarovina et al. 2004; Tsarovina et al. 2010); Insm1 (Wildner et al. 2008); Sox4/Sox11 (Potzner et al. 2010); Isl1 (Huber et al. 2013); Hmx1 (Furlan et al. 2013); Tlx3 (Furlan et al. 2013; Huang et al. 2013)

Sympathetic ganglia are largely dominated by the presence of noradrenergic neurons. As demonstrated by RNA sequencing, sympathetic neurons in mouse thoracic ganglia samples express several genes that are required for the noradrenergic phenotype in a correlated manner, indicating the common regulation of associated gene transcription (Ernsberger et al. 2017). Studies in chick embryos have demonstrated highly correlated gene induction during the differentiation from the neural crest precursors to sympathetic neurons, which is regulated by a common set of TFs (Ernsberger 2000). Studies in mice have partially unraveled the network of TFs involved in this response (Rohrer 2011) (Fig. 1). The coordinated induction of genes contributing to the noradrenergic transmitter phenotype was not observed in cranial parasympathetic neurons. Instead, only incomplete noradrenergic phenotypes, such as TH-expressing cells devoid of DBH, can be observed in the cranial parasympathetic ganglia. A similar differentiation path and neuronal phenotype are rarely observed in the sympathetic ganglia. Occasionally, however, DBH-expressing cells devoid of TH are detected, as was described, in detail, for chick embryos (Ernsberger 2000).

Although the molecular mechanism that drives the segregation of noradrenergic and cholinergic properties in sympathetic neurons is partly understood, the regulation of the cholinergic gene locus in cranial parasympathetic neurons remains largely unresolved, which is also the case for the regulation of NOS expression. In particular, the importance of TFs that are expressed specifically in parasympathetic neurons, such as HMX2 and 3, remains unclear. For HMX1, which is specifically expressed in sympathetic neurons, a critical role in the segregation of noradrenergic and cholinergic properties has been documented, indicating its necessity for TH and TrkA expression (Furlan et al. 2013).

## Mammalian pelvic ganglia and plexus

### Rat pelvic ganglia and the innervation of pelvic organs: noradrenergic and cholinergic/nitrergic neurons with different neuropeptide expression patterns in male and female animals

The pelvic plexus of the male rat includes a large ganglion that is adherent to the lateral lobe of the prostate and receives input from the hypogastric and pelvic nerves. This ganglion encompasses many smaller ganglia that are related to the finer branches of the hypogastric and pelvic nerves (Dail et al. 1975). The large ganglion is referred to as the pelvic ganglion, the “major pelvic ganglion” (MPG) or “anterior pelvic ganglion” (APG) and is generally referred to in studies of male rats.

TH IR cells and CA histofluorescent cells are clustered in the MPG and can easily be distinguished from non-CA ganglion cell clusters (Dail et al. 1975; Arellano et al. 2019). Overall, the abundance of TH-positive cells is low (Warburton and Santer 1993; Ceccatelli et al. 1994; Persson et al. 1998). Quantitative analysis performed in the rat MPG, in combination with neuropeptide analysis, demonstrated that all TH-positive neurons co-express NPY (Keast 1991) and 26.3% of nerve cells in the male rat MPG are TH- and NPY-positive (Keast 1995a) (Table 6).

**Table 6:** Expression of the neurotransmitter synthesizing enzymes TH, DBH, CHAT, and NOS in male and female rodent pelvic neurons

(A) Male rodent pelvic ganglia						
Male MPG/APG	TH+	TH+/ChAT+	TH−/ChAT−	ChAT+	NOS+	
Rat MPG	≈ 33%	0.5%	1 to 2%	2/3		(Keast et al. 1995)
	26%TH+/NPY+					(Keast 1995)
GP APG	22%					(Song et al. 1994)
GP APG	Minority, all NOS−, few DBH−	No		Many, all NOS+	Majority, many CHAT+, some DBH+	(Elfvin et al. 1997)
Mus MPG	34/31%	< 1%	< 5%			(Wanigasekara et al. 2003)
	21%				47.1%	(Yan and Keast 2008)
(B) Female rodent pelvic ganglia						
Female	TH			ChAT	NOS	
Rat PCG	9%					(Houdeau et al. 1995)
Rat AG	20.4%					
Rat HP	12.7%					
Rat MPG	Minority, all ChAT−			Majority, all TH−	Many, all ChAT+	(Persson et al. 1998)
GP PCG	TH	DDC	DBH			
	6–9% all DBH+	≈ 10% TH/DBH+	57 to 73%, many TH−			(Morris and Gibbins 1987)

MPG, major pelvic ganglion; APG, male anterior pelvic ganglion; PCG, female paracervical ganglia; AG, female accessory ganglia; HP, female hypogastric plexus

Proportions of immunopositive cells for the indicated neurotransmitter phenotype markers in rat, guinea pig (GP), and mouse (Mus) pelvic ganglia. Mouse data are obtained for two different mouse strains (see text) and, when the mean numbers between the two strains differ in a notable way, are separated by /. Marker A+ indicates the proportion of cells positive for this marker. Marker A+/marker B+ indicates the proportion of marker B-positive cells double-positive for both markers. Marker A−/marker B− indicates the proportion of cells negative for both markers. The labels “all,” “many,” and “some” in combination with “B−/+” in a column A indicate the proportion of cells positive for the marker A (indicated at the top of the column) that are negative or positive for the marker B. The labels “no,” “minority,” “many,” and “majority” indicate the proportion of cells positive for the respective marker indicated at the top of the column

A small number of TH-positive neurons was observed in the female rat paracervical ganglia (PCG) (Papka et al. 1987; Houdeau et al. 1995). In addition, small, intensively fluorescent cells are present, which become distinguishable from neurons after birth, based on size (Sullivan et al. 1994). In female rats, the nerve cell bodies that innervate pelvic organs are located in the PCG, which constitutes the major ganglion of the female PP, and in smaller ganglia, referred to as accessory ganglia (AG), and the ganglia of the hypogastric plexus (HP)(Houdeau et al. 1995). The proportion of TH-positive neurons differs among these ganglion complexes with 19% in PCG, 20% in AG, and 12% in HN (Table 6).

ChAT-positive cells comprise the majority of neurons in both the male (Keast 1995a) and female (Persson et al. 1998) MPG. TH and ChAT expression are largely mutually exclusive. ChAT-positive cells prevail near or within the penile nerve but are uncommon near the entrance of the hypogastric nerve, where most of the TH-positive cells are found (Keast 1995a). Only 0.5% of the TH-positive cells co-express ChAT, and only 1 to 2% of cells in the MPG express neither TH nor ChAT, indicating that > 98% of the MPG neurons are either noradrenergic or cholinergic (Keast 1995a; Keast et al. 1995). Interestingly, the densities of varicose nerve endings that surround ChAT- and TH-positive somata differ, suggesting that the two neuron populations not only differ in their neurochemistry but also their presynaptic inputs.

Two critical sets of experiments complement these data. The effects of lesioning the hypogastric or pelvic nerves on the innervation of various neuron populations were analyzed in the male rat MPG (Keast 1995b). Lesions in the hypogastric nerve resulted in the loss of innervation for the majority of TH-positive neurons, whereas lesions of the pelvic nerves did not alter the innervation of TH IR somata. Thus, TH-negative and TH-positive neurons differ in the origin of their preganglionic neurons. In another series of experiments, retrograde labeling from the bladder, colon, and penis in male rats demonstrated the highly divergent projection of the different neuron populations in the MPG and AG (Keast and De Groat 1992), as 25 to 30% of neurons labeled from the bladder and colon, but none labeled from the penis, were TH-positive. Neurons labeled from the prostate revealed two populations, 74% of which were TH-positive neurons of large size, and 16% were VAChT-positive cells of small-diameter (Nadelhaft 2003). Retrograde labeling studies performed in the female rat PCG demonstrated that 82% of cells labeled from the urinary bladder were NF200-positive, 22% were NOS-positive, and less than 2% were TH-positive (Forrest et al. 2014). In male rats, the vast majority of neurons (80% to 90%), labeled from penile tissues, were positive for NADPH and NOS (Schirar et al. 1994; Ding et al. 1995; Schirar et al. 1997; Tamura et al. 1997), indicating the importance of nitrergic neurons in the pelvic ganglia.

NOS expression in the pelvic ganglion, which was observed in a large number of neurons, is restricted to cholinergic neurons that co-express VIP but lack TH and NPY (Alm et al. 1995; Persson et al. 1998). Thus, nitrergic neurons in the rat pelvic ganglia constitute a large subpopulation of cholinergic neurons.

In the male rat MPG, VIP expression, which was detected in 44% of neurons, is restricted to cholinergic neurons, located mostly near or in the penile nerve and virtually devoid of NPY (Keast 1991, 1995a; Persson et al. 1998). Interestingly, the vast majority of penis-innervating MPG neurons are VIP- and NOS-positive, whereas retrograde labeling from the bladder and colon revealed that only 5% to 10% of cells were VIP-positive (Keast and de Groat 1989; Ding et al. 1995). Double-labeling confirmed that the vast majority of rat MPG neurons that innervate the penis are VIP- and NOS-positive (Ding et al. 1995). A similar percentage of VIP-positive neurons (46%) can be observed in the female rat PCG; however, 90% of cells in the AG and HP are VIP-positive (Houdeau et al. 1997). Retrograde tracing shows that > 95% of the neurons that project to the myometrium in the lower region of the uterus and cervix are VIP-positive. Importantly, more than 90% of the VIP-positive cells in the female rat PP are NPY-positive, indicating that the uterus is innervated by VIP- and NPY-positive cholinergic neurons. Thus, this subpopulation of VIP/NPY double-positive neurons observed in female animals represents a gender-specific neuropeptide phenotype (Table 7).

**Table 7:** Expression of the neuropeptides VIP and NPY in pelvic ganglia of different rodent species, strains, and sexes and the correlation with neurotransmitter-synthesizing enzymes

(A) Proportions of immunopositive cells for the indicated neuropeptides in rat, guinea pig (GP), and mouse (Mus) pelvic ganglia are provided. Data for mouse are obtained for two different mouse strains (QS, C57). Numbers are given for VIP and NPY-positive cells as well as for VIP/NPY double-positive cells (VIP+NPY+)						
		Ganglion	VIP+	NPY+	VIP+NPY+	
Male						
Rat		MPG	44%	66%	3.7%	(Keast 1995a)
Mus QS		MPG	55%	93%	50%	(Wanigasekara et al. 2003)
Mus C57		MPG	43%	67%	24%	
Female						
Rat		PCG	46%	84%	some	(Houdeau et al. 1997)
		AG	91%	89%	81%	
		HP	89%	94%		
GP		PCG	62 to 65%	72 to 84%	61%	(Morris and Gibbins 1987)
		PCG			60%	(Anderson et al. 1997)
(B) The proportion of VIP and NPY-positive cells among ChAT-positive and -negative as well as NOS-positive cells is provided. The terms “all,” “almost all,” “many,” and “frequently” indicate the size of the respective subpopulation unless given as precise percentages						
	Ganglion	ChAT+/VIP	ChAT−/VIP	NOS+/VIP	NOS+/NPY	
Male rat	MPG	Almost all	3.5%			(Keast 1995a)
Male GP	APG			Many	Frequently	(Elfvin et al. 1997)
Fem. rat	MPG	All				(Persson et al. 1998)
				almost all		(Alm et al. 1995)
fem. GP	PCG	97%		97%		(Anderson et al. 1997)

MPG, male major pelvic ganglion; PCG, female paracervical ganglion; AG, female accessories ganglia; HP, female hypogastric plexus

MPG, male or female major pelvic ganglion; APG, male anterior pelvic ganglion; PCG, female paracervical ganglia

A total of 66% of cells in the male rat MPG are NPY-positive, most of which are TH-negative (Keast 1991; Warburton and Santer 1993; Keast 1995a). In contrast with VIP-positive neurons, NPY-positive neurons are associated with the innervation of the bladder and colon (50% of retrogradely labeled cells were NPY-positive), rather than with penis innervation (5–7% of cells labeled)(Keast and de Groat 1989).

In the female rat PCG, 84% of cells were NPY-positive cells, in addition to 89% of cells in the AG and 94% of cells in the HP. VIP and NPY were co-expressed in more than 90% of VIP-positive cells, unlike in male rats (Houdeau et al. 1997) (Table 7).

Taken together, these data suggest that the pelvic ganglia in the rat are composed primarily of cholinergic neurons, which also, to a large extent, demonstrate nitrergic properties. The small population of noradrenergic neurons appears to differ in prevalence between male and female animals and they do not show the co-expression of nitrergic features. In male and female animals, the neuropeptide VIP appears to be restricted to cholinergic/nitrergic cells. The neuropeptide NPY is co-expressed with TH but is not restricted to noradrenergic neurons. In particular, the female AG displays a large number of ChAT/VIP/NPY-positive neurons, which appear to represent a gender-specific neuron population. Target-specific characteristics were also observed, with cholinergic, nitrergic, and VIP-positive cells innervating the penis rather than the bladder and colon.

### The guinea pig pelvic ganglia: determining differences in TH and DBH expression

The distribution of CA histofluorescence observed among pelvic autonomic neurons revealed a mixture of fluorescent and non-fluorescent ganglia, starting within the hypogastric nerve (HN) and extending into groups of fluorescent and non-fluorescent cells, in the anterior (APP) and posterior (PPP) pelvic plexus of adult guinea pigs (Furness and Costa 1973). The quantification of the paracervical ganglia in adult female guinea pigs showed that, on average, 6% of the neurons in the ganglia (2% to 18% in different ganglia) displayed CA histofluorescence (Morris and Gibbins 1987). Correspondingly, cells expressing TH and dopa decarboxylase (DDC), which are enzymes in the noradrenaline biosynthesis cascade that precede DBH, represent 6 to 10% of cells. An increased number of TH-positive cells is observed in the adult male pelvic ganglia (Song et al. 1994) (Table 6). TH and NOS expression are mutually exclusive (Elfvin et al. 1993), with TH-positive cells occurring preferentially in the anterior part of the ganglion, whereas NOS-positive cells are primarily located in the caudal region (Elfvin et al. 1997).

Although TH-expressing neurons are also DBH-positive, supporting their noradrenergic phenotypes, many DBH-positive cells exist that do not express TH (Dhami and Mitchell 1991; Elfvin et al. 1993; Morris et al. 1997). Indeed, the majority of neurons in the female PCG (57 to 73%) express DBH and NOS but are TH-negative (Morris and Gibbins 1987; Dhami and Mitchell 1991; Elfvin et al. 1993) (Table 6). Less than 1% of TH-positive cells in guinea pig co-express VIP (Song et al. 1994).

Nitrergic neurons that co-express ChAT represent a substantial number of neurons in the male pelvic ganglion (Elfvin et al. 1997). NOS was frequently found to be co-expressed with VIP and NPY, with some neurons containing both (Song et al. 1994; Elfvin et al. 1997). Groups of NOS-positive cells were also positive for SOM, and some co-expressed CGRP. The co-expression of TH and VIP has not been reported; however, the majority of TH-positive cells are also NPY-positive.

In the female PCG, VIP is co-expressed with ChAT and NOS (97%), but not with TH (Morris and Gibbins 1987; Song et al. 1994; Anderson et al. 1997) (Table 7). NPY is expressed in the majority of female PCG neurons (approximately 80%), and more than half of these co-express VIP (Morris and Gibbins 1987). Thus, similar to the situation observed in the rat, a significant number of female pelvic neurons co-express the neuropeptides VIP and NPY.

A detailed analysis of the expression of the neuropeptides VIP, NPY, and SOM and the transmitter-synthesizing enzymes TH, ChAT, and NOS was used to define 11 classes of neurons in the female PCG, with fewer than 10% of these being classified as noradrenergic neurons (Morris and Gibbins 1987). In combination with the IHC properties of the terminal synaptic baskets, which surround the characterized neuron classes, specific connections could be demonstrated. Almost all noradrenergic neurons appear to be surrounded by NPY-positive terminals (Morris and Gibbins 1987). In contrast, most of DBH/NPY/VIP-positive cell bodies are surrounded by baskets of substance P-positive nerve fibers. Dense baskets of enkephalin (ENK)-positive terminals surround cell bodies containing SOM alone, indicating that each of these relatively small (generally less than 10% of ganglion cells) subpopulations is wired to a specific circuit, from pre-ganglionic to post-ganglionic neurons. Using the same approach, a connectivity pattern for male guinea pig APP neuron classes has been established (Dhami and Mitchell 1991).

Taken together, the studies performed on the guinea pig pelvic ganglia confirmed the small size of the noradrenergic neuron population, with a difference observed between the sexes, as described in rats. In addition, these studies demonstrated the uncoupling between TH and DBH expression. Unlike the cranial parasympathetic ganglia, in which TH and DBH are not co-expressed, in the rodent pelvic ganglia, the number of DBH-positive cells largely outnumbered TH-positive cells. The non-noradrenergic cells mostly showed the co-expression of cholinergic and nitrergic features, with a negligible percentage of neurons that were non-noradrenergic/non-cholinergic or of a mixed phenotype. Similar to rats, a large population of neurons that co-express the neuropeptides VIP and NPY were found in female animals.

### The autonomic neurons of the human pelvic plexus: an enlarged noradrenergic subpopulation and developmentally regulated NOS co-expression, in both noradrenergic and cholinergic neurons

In intra-pelvic soft tissue preparations, obtained from adult body donors, pelvic ganglion cells were found surrounding nerve bundles, in enlargements of nerve bundles, in round or oval ganglia, surrounded by a connective tissue capsule, and within the structures of numerous intermediate morphologies (Takenaka et al. 2005). The strict definition of a ganglion proved to be difficult, and the term “ganglion cell cluster” was preferred (Takenaka et al. 2005; Muraoka et al. 2018). Along the pelvic splanchnic nerves, ganglion cells could be found, and also the hypogastric nerve contained ganglion cell clusters, including a large ganglion along its distal course. Unlike in rodents, where the pelvic ganglion cells are found preferentially in the major pelvic ganglia, human pelvic ganglion cells are distributed over a larger area and in various locations. Similar to experimental rodents, however, the prevalence of TH-positive cells changes dramatically, from an average of 90% of cells in the sacral sympathetic ganglia to 58% in the HN and PP, and 36% in the pelvic splanchnic nerves (Takenaka et al. 2005).

Overall, more than half of the neurons in the human PP appeared to be TH-positive (Takenaka et al. 2005; Imai et al. 2006) (Table 8). Adjacent to the bladder neck, the proportion of TH-positive neurons was reduced (45%) compared with that near the prostate (67%), in male postnatal donors between 2 and 12 months of age (Jen et al. 1996a). Importantly, all TH-expressing cells were DBH-positive, indicating a noradrenergic transmitter phenotype. VAChT-positive cells in male infants, from 2 months to 3 years of age (Dixon et al. 1997,2000), represented approximately 40% of all neurons. Comparable with rodents, the vast majority of neurons appeared to be either noradrenergic or cholinergic but not mixed. In the pelvic ganglia close to the bladder neck and prostate of male neonates and children, only 8% of the cells co-expressed the noradrenergic marker TH and the cholinergic marker VAChT (Dixon et al. 1999).

**Table 8:** Expression of neurotransmitter-synthesizing enzymes and neuropeptides in the neurones of the human pelvic plexus

(A) The proportions of cells immunopositive for TH, VAChT, both markers (TH/VAChT), or NOS. Numbers are listed according to age for adults (39 to 85 years) as well as neonates, infants, and children (2 months to 7 years)							
Donor age		Site	TH	TH/VAChT	VAChT	NOS	
72–85 years		PP	58%				(Takenaka et al. 2005)
Mean 79 years		PP	> 50%				(Imai et al. 2006)
64–82 years		PP				> than TH	(Muraoka et al. 2018)
39–77 years		PP DD SV				Variable	(Grozdanovic and Goessl 1999)
7 weeks–6 years		PP, male		8%			(Dixon et al. 1999)
6 months–7 years		PP, male			40%	65%	(Dixon et al. 2000)
2 to 12 months		AB	45%				(Jen et al. 1997)
		AP	67%				
(B) The proportion of TH-positive, TH-negative, and VAChT-positive cells (co)expressing NOS in human pelvic plexus ganglia of infants and children							
		NOS/TH+	NOS/TH −	NOS/VAChT+			
AB		61%	77%				(Jen et al. 1996a)
AP		38%	59%				
PP				65%			(Dixon et al. 2000)
(C) The expression of NPY and VIP in TH-positive and -negative as well as VAChT-positive human pelvic plexus neurones in infants and children							
	NPY	NPY/VAChT	VIP/TH+	VIP/TH−	NPY/TH+	NPY/TH−	
AB			64%	83%	66%	92%	(Jen et al. 1996a)
AP			42%	82%	62%	65%	
PP	85%	Almost all					(Dixon et al. 2000)

PP, pelvic plexus; PP DD SV, pelvic plexus ganglion cell clusters near ductus deferens and seminal vesicles; AB, extramural ganglia adjacent to the bladder; AP, extramural ganglia adjacent to the prostate

> than TH: the proportion of NOS-positive cells exceeds that of TH-positive cells; variable: highly divergent proportions of NOS-positive cells

AB, adjacent to bladder; AP, adjacent to prostate gland; PP, pelvic plexus

The majority of TH-positive as well as TH-negative neurons in infants and children pelvic plexus ganglia express NPY and VIP. “Almost all” VAChT-positive neurons express NPY. The situation in adults and the comparison between males and females could not be retrieved from PubMed

In agreement with the segregation of noradrenergic and cholinergic markers in plexus neurons, TH and VAChT IR was detected in separate nerve fibers in two important male pelvic target tissues, the corpus cavernosum and corpus spongiosum (Hedlund et al. 2000). This segregation corresponds with the segregation between TH- and NOS-positive cells, and the co-expression of VAChT, NOS, and VIP in fibers and terminals innervating these target tissues. In nerve fibers that innervate the vas deferens, examined in 28- to 83-year-old males, TH and DBH are co-expressed with NPY, but lack VAChT (Jen et al. 1999). Remarkably, during childhood stages, fibers that innervate the seminal vesicles and vas deferens may show NOS expression in both TH-positive and TH-negative fibers (Jen et al. 1997). These data indicated that pelvic nerve fibers and neurons in the PP ganglia in humans may show NOS expression in both noradrenergic and cholinergic units, possibly regulating this co-expression during development.

Reportedly, in the pelvic ganglion cell clusters of adult human donors, NOS-positive neurons have been consistently observed at higher abundance than TH-positive cells (Muraoka et al. 2018). TH-positive neurons were found to not express NOS and VIP. Approximately 20% of cells in the adult human PP ganglia, near the junction of the vas deferens and seminal vesicle, were reported as NOS-positive, suggesting that different proportions of NOS-positive cells may be found in different plexus regions (Grozdanovic and Goessl 1999).

As indicated previously, age-dependent changes in expression must also be considered. The quantification of pelvic tissues obtained from childhood stages (6 months to 7 years) showed that 65% of neurons were NOS-positive (Dixon et al. 2000) (Table 8). In tissue derived from male infants and children, TH and NOS are abundantly co-expressed in fibers that innervate diverse targets, such as the vas deferens, seminal vesicles, the prostate, and the bladder neck (Jen et al. 1996b). Ganglia adjacent to the bladder neck included 61% of neurons that were TH/NOS double-positive, whereas this proportion in ganglia adjacent to the prostate was 38% (Jen et al. 1996a). Of the approximately 40% of cells in the pelvic ganglia that were VAChT-positive in male infants, 65% coexpressed NOS (Dixon et al. 1997, 2000). TH/NOS-positive fibers in infants and children can be found in the bladder neck, ureter, and prostate, and the vas deferens and seminal vesicles are supplied by a dense TH/NOS-double-positive fiber net (Jen et al. 1996b). Most remarkably, the coexistence of TH with NOS (and VIP) becomes lost in adults, as observed for the vas deferens (Jen et al. 1999). The moderate innervation in adults by NOS-, VIP-, and VAChT-positive neurons, rather than TH-positive neurons, requires a currently unknown cellular transdifferentiation or reorganization mechanism for pelvic organ innervation and/or cell type re-specification.

In pelvic ganglion cell clusters from adult human donors, the co-expression of NOS with VIP can be detected (Muraoka et al. 2018). In infants and children, a large extent of co-expression between VIP has been reported for both NOS and TH (Jen et al. 1996a) (Table 8). In the ganglia adjacent to the bladder neck of male infants and children, 64% of the TH-positive cells (and 83% of the TH-negative cells) co-express VIP, as do 42% of TH-positive cells (vs. 82% of TH-negative cells) in the ganglia adjacent to the prostate (Table 8). For adult penile innervation, 50% of the perivascular nerve fibers in the corpus cavernosum and spongiosum and 90% of the trabecular nerve fibers are NOS- and VIP-positive (Ehmke et al. 1995). In the fibers of the corpus cavernosum and corpus spongiosum, VIP IR has been associated with VAChT expression, in addition to NOS expression, but not with TH expression (Hedlund et al. 2000).

Approximately 85% of the neurons in the pelvic ganglia proximal to the bladder and prostate in male neonates and infants express the neuropeptide NPY (Dixon et al. 2000) (Table 8). The co-expression of TH and NPY can be detected in 66 and 62% of the cells in the ganglia adjacent to the bladder neck and the prostate, respectively (Jen et al. 1996a). Similar to rodents, the expression of NPY in human pelvic neurons is not restricted to noradrenergic neurons, as 92 and 65% of TH-negative cells co-express NPY in the ganglia adjacent to the bladder neck and the prostate, respectively (Table 8). Of the approximately 40% of cells in the pelvic ganglion of male infants that are VAChT-positive, almost all of them co-express NPY (Dixon et al. 1997, 2000). In a study that compared the expression of diverse neuropeptides, including NPY and VIP, in the nerves that innervate the penis in 21- to 34-year-old male donors (Hauser-Kronberger et al. 1994), a qualitatively similar distribution of these two peptides was observed among blood vessels, sinusoids, and trabeculae in the cavernous and spongious bodies, glans, and urethra. NOS-positive cells that co-express NPY and TH can be observed in the vas deference and seminal vesicles, in children (Jen et al. 1997).

When comparing the distribution of neurotransmitter phenotypes among pelvic neurons, key differences between humans and rodents have become apparent, raising several questions. One such quantitative difference in neuronal subpopulation compositions concerns the proportion of noradrenergic neurons in the human PP (more than 50%), which significantly exceeds that in the rodent pelvic ganglia (10 to 30%, depending on sex). Whether the gender-specific differences in peptide expression patterns that have been observed in rodents also exist in humans remains to be seen. Finally, changing co-expression patterns, in terms of the noradrenergic and nitrergic properties, which have been observed in human target tissues, also raises a wide range of highly relevant questions. Is such a shift also observed in rodents and can it be mechanistically explored in experimental animals? Does this shift occur due to changes in gene expression patterns in individual cells or due to the maturation of fiber connections, in possible combination with the expansion or restriction of defined neuron subpopulations? How does this shift contribute to changes in the activity patterns of pelvic organs during aging or disease and can this be modulated therapeutically?

### Neurotransmitter phenotypes observed in intramural ganglia of the mammalian bladder

The histology and function of intramural ganglia in the bladder were investigated in the guinea pig. In newborn animals, numerous intramural ganglia were detected, lying among smooth muscle bundles and in the submucosa (James and Burnstock 1988). The small number of neurons that expressed DBH (1 to 6%) and the scarcity of TH-positive cells indicated that the noradrenergic neuron population in the guinea pig intramural ganglia is likely of little importance (James and Burnstock 1988; Smet et al. 1996b; Werkstrom et al. 1998). Instead, NADPH-reactive and NOS-positive neurons were abundant, distinct from TH-positive cells, indicating the importance of nitrergic signaling (Saffrey et al. 1994; Smet et al. 1994). Different studies have reported that between 50 and 70% of cells in the intramural ganglia are NOS-positive (Smet et al. 1996b; Werkstrom et al. 1998; Zhou and Ling 1998). NPY can also be detected in each of these ganglia, in 70 to 85% of the neurons (James and Burnstock 1988). The prevalence of this peptide has also been described in other studies; however, the reports regarding the prevalence of other peptides diverge (Crowe et al. 1986; James and Burnstock 1988; Smet et al. 1996b; Werkstrom et al. 1998; Zhou and Ling 1998).

Similarly, in the intramural ganglia of the bladder in adult humans, NOS-positive cells have frequently been detected, constituting 72 to 96% of the neuronal subpopulation (Smet et al. 1996b; Smet et al. 1996a) (Table 9). TH-positive neurons can be observed only occasionally, representing 14% of the neuron population, based on tissue samples taken from patients undergoing cystectomy for bladder carcinomas or prostatectomy for prostate cancer (Smet et al. 1996a, 1996b). In a sample of patients with bladder instability and diverse voiding problems, no CA histofluorescence-positive neurons were detected, but the ubiquitous expression of acetylcholinesterase was considered to be an indication of a ubiquitous cholinergic transmitter phenotype (Dixon et al. 1983).

**Table 9:** Expression of neurotransmitter-synthesizing enzymes and neuropeptides in intramural ganglia of human bladder

(A) The proportions of neurones immunopositive for the neurotransmitter phenotype markers NOS, TH, DBH, and VAChT. Numbers are provided for adult tissue obtained from cancer patients and neonates, infants, and children (at 2 months to 6 years of age) dying from sudden child’s death (CD) or accidental trauma (AT)						
Age	Death	% NOS	NOS/TH+	NOS/TH−	NOS/VAChT	
Adult	Cancer	86%				(Smet et al. 1996b)
7 weeks–6 years	CD, AT				40%	(Dixon et al. 2000)
2 months–3 years			58%	45%		(Dixon et al. 1997)
(B) The proportion of neurons immunopositive for noradrenergic and cholinergic markers in intramural ganglia of adult humans as compared with neonates and children						
Age	Death	TH	TH+DBH+	TH+VAChT+	VAChT	
Adult	Cancer	14%				(Smet et al. 1996b)
7 weeks–6 years	CD, AT				75%	(Dixon et al. 2000)
7 weeks–6 years	CD, AT		25% no VAChT	50%	25% no TH	(Dixon et al. 1999)
2 months–3 years	CD, AT	40%				(Dixon et al. 1997)
(C) The proportion of neurons immunopositive for the neuropeptides VIP, NPY, CGRP, SOM, and GAL						
Age	VIP	NPY	CGRP	SOM	GAL	
Adult	77%	58%	0%	0%	65%	(Smet et al. 1996b)
2 months–3 years	45% TH−	90% TH−	65% TH−	90% TH−		(Dixon et al. 1997)
	40% TH+	70% TH+	54% TH+	73% TH+		

%: The overall proportion of positive cells in intramural ganglia. NOS/marker B−/+ indicates the proportion of NOS-positive cells among marker B-negative or positive cells. In infants and children, approximately half of TH-negative as well as -positive express NOS. The corresponding numbers for adults are not determined

Whereas 40% of the cells express TH in neonates and children, their proportion drops to 14% in adults. Coexpression of TH is observed with DBH or VAChT

Massive changes are observed in neuropeptide expression patterns between infants and adults. The proportion of VIP-positive cells increases and of NPY-positive cells decreases with age. Particularly striking appear the loss in CGRP and SOM expression

Further studies disclosed a noradrenergic component in the intramural ganglia, depending on age (Table 9). In samples obtained from infants aged 2 months to 3 years, and from children who died due to accidental trauma, 40% of cells were found to be TH-positive, compared with 60% that were TH-negative (Dixon et al. 1997). Approximately 25% of all neurons co-expressed TH and DBH, although a large proportion of TH-positive cells lacked DBH (Dixon et al. 1999). In samples of the intramural ganglia of the human bladder neck and trigone from male infants and children, 25% of the neurons expressed TH alone, 50% expressed both TH and VAChT, and 25% expressed VAChT alone (Dixon et al. 1999). These data indicated that a significant number of TH-positive and CA cells during the infant and childhood stages, which are normally reduced during adulthood appears affected in people with bladder dysfunction. This developmental change differs from that observed for the proportion of NOS-positive cells in the intramural ganglia (Table 9). The proportion of TH-positive neurons in adults decreases to little more than 10%, whereas the proportion of NOS-positive neurons increases, from approximately half of the neurons during childhood to almost 90% of neurons in adults (Smet et al. 1996b; Dixon et al. 1997; Jen et al. 1997; Dixon et al. 2000). These data indicated the existence of a strong shift from noradrenergic to nitrergic neurotransmission in the human intramural bladder ganglia with increasing age.

A shift in the neuropeptide expression pattern has also been observed from childhood to adulthood (Table 9). During this transition, the proportion of NPY-positive neurons decreases, from 70% of TH-positive and 90% of TH-negative cells to an overall abundance of approximately 60% of all cells in adults (Smet et al. 1996a; Dixon et al. 1997). In parallel, the abundance of VIP-positive neurons increases from approximately 40% to close to 80%. Remarkably, the proportion of SOM-positive neurons decreases, from approximately 70% of TH-positive and 90% of TH-negative neurons in infants and children to the complete absence in adults (Smet et al. 1996a; Dixon et al. 1997). Likewise, the proportion of CGRP-positive neurons decreases, from approximately 60% of neurons in infants and children to the complete absence in adults. These results indicate extensive changes in the neuromodulatory roles played by neuropeptides in the intramural ganglia during the transition to adulthood.

### Composition and development of the mouse pelvic ganglia

In a comparative analysis of two different mouse strains, inbred C57BL/6 and outbred Quackenbush–Swiss (QS), the neuron subpopulation composition in the pelvic ganglia of mice was observed to be similar but not identical to those in other rodents (Wanigasekara et al. 2003). The proportion of noradrenergic neurons was 31 and 34%, with almost complete coexistence between TH and DBH expression. DBH-positive, TH-negative cells were only rarely observed. TH and ChAT expression are complementary, with fewer than 1% of cells found to be double-positive. Fewer than 5% of cells are neither TH- nor ChAT-positive, which indicates that noradrenergic and cholinergic gene expression is almost completely segregated and only a very small cell population is both non-noradrenergic and non-cholinergic. The proportion of cholinergic neurons was calculated to be approximately 60%. Approximately 50% of the cells in the two mouse strains express VIP. Importantly, less than 1% of the TH-positive neurons co-express VIP, but all TH-positive cells co-express NPY, and a significant number of TH-negative, cholinergic cells also co-expressed NPY. Clusters of VIP-positive neurons were detected near the origin of the penile nerve, within the origin of this nerve, and some distance (1 mm from the ganglion) further distally.

The neuron numbers in the mouse pelvic ganglion were determined based on Hu staining (Yan and Keast 2008), which indicated the post-natal doubling of the total neuron population. NOS and TH cells form different populations of Hu-positive neurons at P0 compared with those in adults. During this time, ganglion composition changes from P0 (17.5% NOS and 23.9% TH-positive, 61.5% NOS- and TH-negative) compared with the adult (47.1% NOS-positive, 29.1% TH-positive, and 27.3% NOS- and TH-negative). These changes may be due to the large increase in the density of NOS-positive neurons and a smaller increase in TH-positive neurons. No significant changes were observed for the double-negative cells. The major increase in NOS-positive cells was observed between P0 and P7, and the cell numbers reach and maintain a plateau through P14, followed by a smaller increase from P14 to P21. In contrast, the adult TH-positive numbers are achieved by P7. The production of new neurons occurs due to neuronal precursor cells, which are present at P0, as characterized by the expression of p75 and the absence of Hu/S 100.

The development of TH and NOS innervation in urogenital organs was analyzed in male mice (Yan and Keast 2008). In the corpus cavernosum tissue, at P7, axons are observed at a modest density and fibers immunoreactive for NOS more numerous than those for TH. At P14, axons appear to be more numerous but are found at similar relative densities. In adults, the TH-positive fiber prevalence is similar to greater than that of NOS-positive fibers. In the bladder muscles, at all postnatal stages, NOS fibers are much more numerous than TH fibers.

With the help of the Venus reporter transgene, under the control of the Sox10 promoter, the emigration of sacral neural crest cells and the population of the pelvic viscera has been reported (Wiese et al. 2017). By E10.5, lumbosacral neural crest cells migrate ventrally between the neural tube and somites. At this early stage, neuronal differentiation can be detected by HuC/D and TUJ1 staining. Between E10 and E11, sacral neural crest cells delaminate and emigrate, both laterally and ventrally, as a loose stream of cells. By E12.5, a definitive pelvic ganglion becomes visible, and the pelvic plexus becomes populated by neural crest stem cells that travel along the nerves coming from the spinal cord and the dorsal root ganglia (DRG). By E14.5, glial and neuronal differentiation commences in the pelvic ganglion. In addition to the formation of the pelvic ganglion, the Sox10 signal can be detected in discrete cells within the bladder body and urethra, at E15.5 (Wiese et al. 2012). By E15.5, TH and VAChT expression can be observed by IHC, in the region of the pelvic ganglion. Comparisons of the genome-wide TF expression in whole-mount mouse embryos (Gray et al. 2004) revealed the expression of a set of TFs in the pelvic ganglia at E15.5 (Wiese et al. 2012). These TFs include Phox2b, Hand1 and 2, Gata2 and 3, and HMX1, which are critically and specifically involved in sympathetic neuron development.

**Fig. 2:**
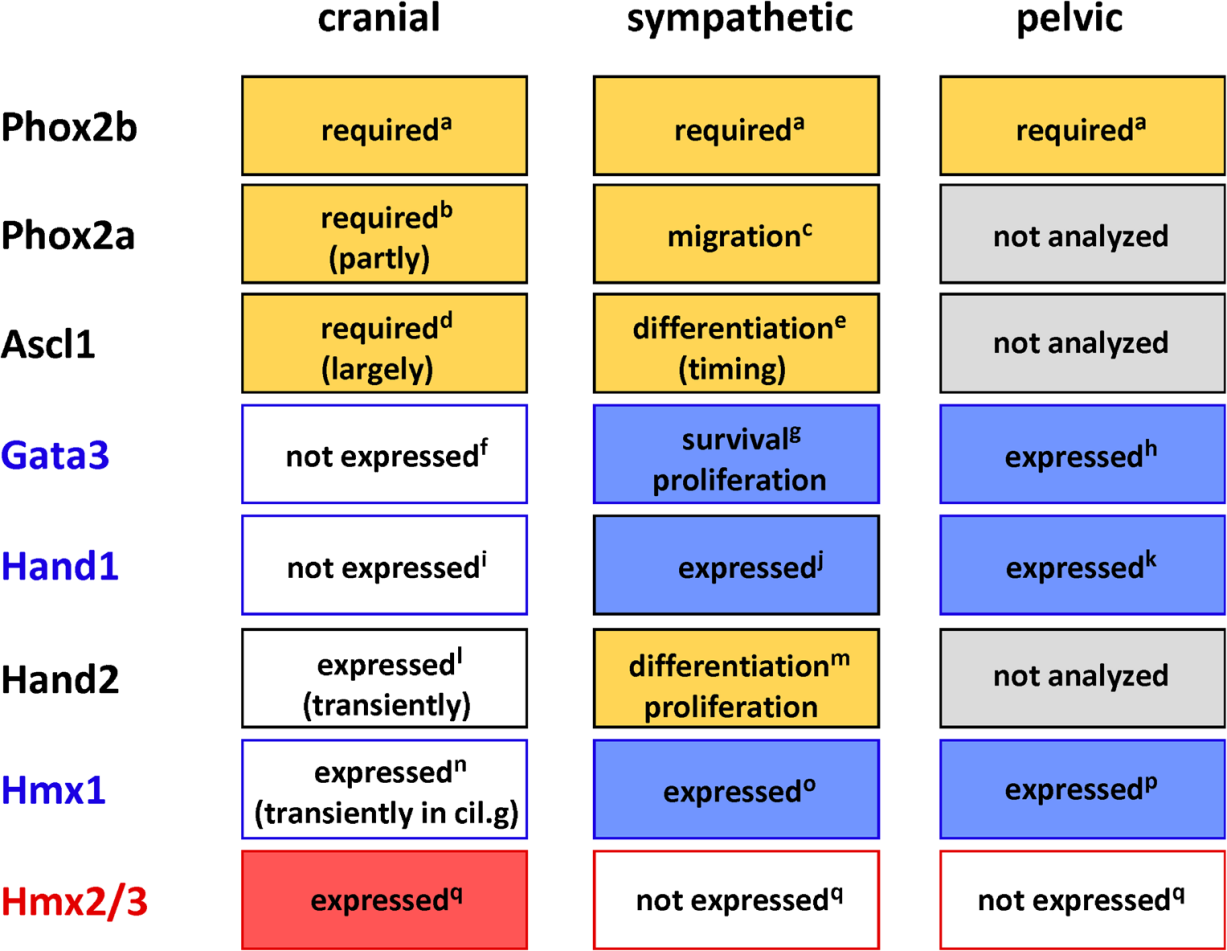
Transcription factors in autonomic neuron differentiation. Transcription factors detected during embryonic differentiation of autonomic postganglionic neurons in mice are shown for cranial, paravertebral, and pelvic ganglia. Their function, as far as they are characterized, are indicated and discussed in the text. The basic helix-loop-helix protein Hand 1 and the zinc finger protein Gata 3 are selective markers for sympathetic and pelvic neuron development. The H6 family homeobox protein homologues HMX 2 and 3 are selective markers for cranial postganglionic neurons and distinguish parasympathetic from the sympathetic and pelvic neuron lineages. ^a^(Pattyn et al. 1999); ^b^(Morin et al. 1997; Pattyn et al. 1997); ^c^(Coppola et al. 2005); ^d^(Hirsch et al. 1998); ^e^(Pattyn et al. 2006); ^f^(Espinosa-Medina et al. 2016); ^g^(George et al. 1994; Lim et al. 2000; Tsarovina et al. 2004; Moriguchi et al. 2006; Tsarovina et al. 2010; Espinosa-Medina et al. 2016); ^h^(Espinosa-Medina et al. 2016); ^i^(Espinosa-Medina et al. 2016); ^j^(Doxakis et al. 2008; Espinosa-Medina et al. 2016; Firulli et al. 2017), ^k^(Espinosa-Medina et al. 2016); ^l^(Müller and Rohrer 2002; VanDusen et al. 2014; Stanzel et al. 2016); ^m^(Howard et al. 1999; Howard et al. 2000; Lucas et al. 2006; Morikawa et al. 2007; Hendershot et al. 2008; Schmidt et al. 2009; Vincentz et al. 2012); ^n^(Wang et al. 2000); ^o^(Yoshiura et al. 1998; Furlan et al. 2013); ^p^(Wiese et al. 2012); ^q^(Espinosa-Medina et al. 2016)

A landmark finding was the observation that the expression of the Hand1 and Gata3 TFs correlated with the absence of HMX2 and 3 during pelvic ganglion development (Espinosa-Medina et al. 2016). HMX2 and 3 are TFs that are specifically expressed during the development of cranial parasympathetic post-ganglionic neurons, whereas Hand1 and Gata3 constitute markers for sympathetic neuron development (Fig. 2). In addition, interference with motor fiber outgrowth from the sacral spinal cord does not compromise the formation of the pelvic ganglion anlage, indicating that ganglion formation and cell differentiation does not depend on the nerve-associated Schwann cell precursors, unlike the cranial parasympathetic ganglia. In addition, pelvic ganglia form prior to innervation, and Phox2b is not expressed in Sox10-positive cells associated with pelvic ganglion innervation. These eminent developmental features have resulted in the conclusion that the pelvic ganglion should be classified as a “sympathetic” ganglion (Espinosa-Medina et al. 2016).

The development of a fluorescence-assisted cell sorting (FACS) strategy for the identification of autonomic precursors in visceral tissues (Buehler et al. 2012) and the genetic targeting of precursor and differentiating neuron subpopulations have made analyzing the differentiation processes in rodents possible, starting from the characterization of neural crest precursors to the analysis of diverse pelvic neuron subpopulations. In particular, the segregation of noradrenergic and cholinergic neurons that operate in the pelvic domain will be of interest. The regulation of NOS expression poses another critical topic. The restriction of different neuropeptides to noradrenergic and cholinergic neuron subpopulations also remains an open question.

## Concluding Remarks

### Distribution of different peripheral autonomic neuron populations along the body axis

**Fig. 3:**
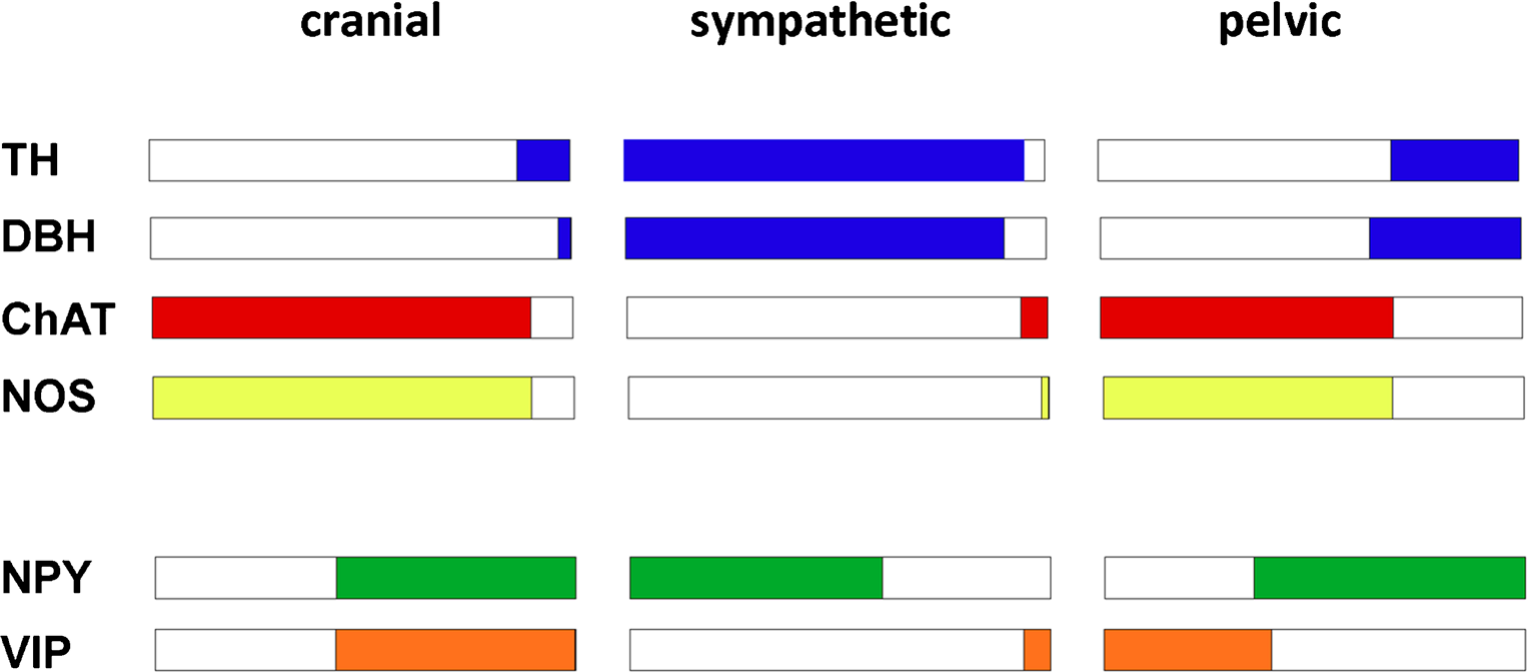
Presence of the classical neurotransmitters noradrenaline and acetylcholine, the small molecule neuromodulator nitric oxide and the neuropeptides NPY and VIP in cranial autonomic, sympathetic, and pelvic ganglia. The length of the bars indicate the proportion of neurons positive for the respective marker. The horizontal position indicates presence in cranial, sympathetic, and pelvic ganglia as well as their co-expression with other markers. Both proportion and co-expression are detailed in the manuscript text and tables. Data shown are mainly from rat. As for rat pelvic ganglia, quantification of the proportion of DBH-positive cells is not available; data from the guinea pig are shown

Key landmarks in the organization of the peripheral autonomic neurons include the prevalence of cholinergic and nitrergic properties in the cranial parasympathetic domain, the prominence of noradrenergic properties in the paravertebral and prevertebral sympathetic ganglia, and the mixture of these features in the pelvic region (Fig. 3). Remarkably, a unique combination of TFs can be observed in the embryonic cranial parasympathetic ganglia, while a distinguished TF fingerprint unifies sympathetic and pelvic neurons during mouse development (Fig. 2).

In addition to the specific cranial parasympathetic TF fingerprint, which encompasses the H6 homeobox family TFs HMX2 and 3 and the basic helix loop helix TF ASCL1, the cranial autonomic neurons are generated from different precursor cells, the Schwann cell precursor-like progenitors. During differentiation, these cells generate cholinergic and nitrergic neurons; however, they appear to be unable to realize a noradrenergic transmitter phenotype, relying on the coordinated expression of enzymes necessary for the synthesis of noradrenaline, together with vesicular and plasma membrane monoamine transporters. Surprisingly, two key enzymes, TH and DBH, can be expressed in the domains of the developing cranial ganglia, but are expressed separately and in an uncoordinated manner. A large question that remains is how the expression of the cholinergic locus and NOS genes are addressed during the development of these neurons.

The coordinated expression of the features that characterize the noradrenergic neuronal phenotype is typical for sympathetic neuron development. However, this development appears to begin with the co-expression of the cholinergic locus, in sympathetic progenitors and neuroblasts. How this process is orchestrated by the known TFs, including Phox, Hand, Gata, HMX, and Insm TFs, remains unclear. Chromatin modifications and the integration of gene loci into gene regulatory circuits during the transition from a neural crest precursor to a differentiated sympathetic neuron are potential mechanisms. How this process is refined, mechanistically, how it is spatially coordinated during the segregation of noradrenergic and cholinergic features, and how this process compares with the processes identified in cranial parasympathetic neurons remain key questions that must be answered.

The development of the pelvic nervous system in mice depends on the same TFs as the development of the sympathetic nervous system; therefore, similar questions must be answered for the sympathetic and pelvic nervous systems. How many neural crest-derived progenitors become mixed noradrenergic/cholinergic immature neurons? Which processes regulate the segregation of noradrenergic and cholinergic features and result in the diverse ratios of the two subtypes observed in different mammalian groups? What processes govern the co-expression or segregation of nitrergic features with either of the other two primary transmitter phenotypes? How is the expression of small-molecule neurotransmitter phenotypes coordinated with neuropeptide expression and which roles are played by target tissues during these processes? Finally, the presence of gender-specific neuron populations and their distribution and generation remains an unresolved topic.

The diversity of autonomic neurons is apparent in the differential co-expression of neuropeptides with the classical noradrenergic, cholinergic, and nitrergic transmitter phenotypes. These various patterns equip individual neurons with specific signaling fingerprints, often referred to as neurochemical codes. A critical observation was made during rat postnatal development, in which the cholinergic sweat gland-innervating neurons appeared to acquire their mature neurotransmitter phenotypes by switching from a noradrenergic to a cholinergic phenotype, co-expressing the neuropeptide VIP under the influence of target-derived growth factors. This finding shaped the concept of critical neuron–target interactions, which may be responsible for the final maturation of innervating neuron properties and may reflect the co-expression of classical neurotransmitters and neuropeptides in target-specific combinations. Target-dependent differentiation has subsequently been described for two noradrenergic neuron subtypes that innervate the piloerector and nipple erector muscles.

The observation that VIP can be detected in cholinergic sympathetic neurons that innervate the sweat glands of rats (Landis and Fredieu 1986), cats (Lindh et al. 1989; Anderson et al. 1995), and humans (Schulze et al. 1997; Donadio et al. 2019) revealed the first example of the conservation of a transmittal signaling code across multiple mammalian orders. This signaling code includes a sweat gland–derived neurokine, which acts as a differentiation factor through the activation of the LIFRβ/gp130 receptors in mice and humans (Stanke et al. 2006; Di Leo et al. 2010; Melone et al. 2014). In addition, the finding that NPY is absent from noradrenergic piloerector sympathetic innervating neurons in guinea pigs (Gibbins 1992), mice (Furlan et al. 2016), and humans (Donadio et al. 2019) strengthens the concept that target-specific autonomic pathways are characterized by a conserved signaling code, composed of a classical neurotransmitter, with or without a distinguishing neuropeptide. An interesting exception was detected in non-noradrenergic, VIP-positive sympathetic fibers directed toward guinea pig (Gibbins 1992) and cat (Lindh et al. 1989; Anderson et al. 1995) blood vessels in muscles that are considered to act as muscle vasodilators. Similar fibers cannot be detected in rats and mice (Guidry and Landis 2000).

Taken together, this wealth of data indicates the existence of conserved and non-conserved autonomic neurotransmitter/modulator codes along the rostrocaudal body axis and for target-specific efferent pathways. The mechanisms through which target-specific autonomic neural circuits are established during development and connect pre-ganglionic with post-ganglionic neurons and their target tissues are incompletely understood. The exploration of the full transcriptomes of these diverse neuron populations and individual neurons is likely to provide insights into the molecular players that may be involved in these processes.

### Integration of autonomic neuron subpopulations into target-specific efferent pathways

Comparative analysis of the reflex changes in sympathetic neuron activity in response to various stimuli has demonstrated the selective regulation of distinct target-specific neuronal pathways to be a key factor in the neural regulation of homeostasis (Jänig 2006). Through the characterization of full transcriptomes in different autonomic neuron populations, using RNA sequencing methods (Furlan et al. 2016; Zeisel et al. 2018), the molecular fingerprints of neuronal diversity have also revealed expression of the molecular players potentially involved in the generation of target-specific pathways, synapse formation, and stabilization, and promise to shed light on the molecular mechanisms underlying their integration into target-specific efferent pathways (Ernsberger 2019).

**Fig. 4:**
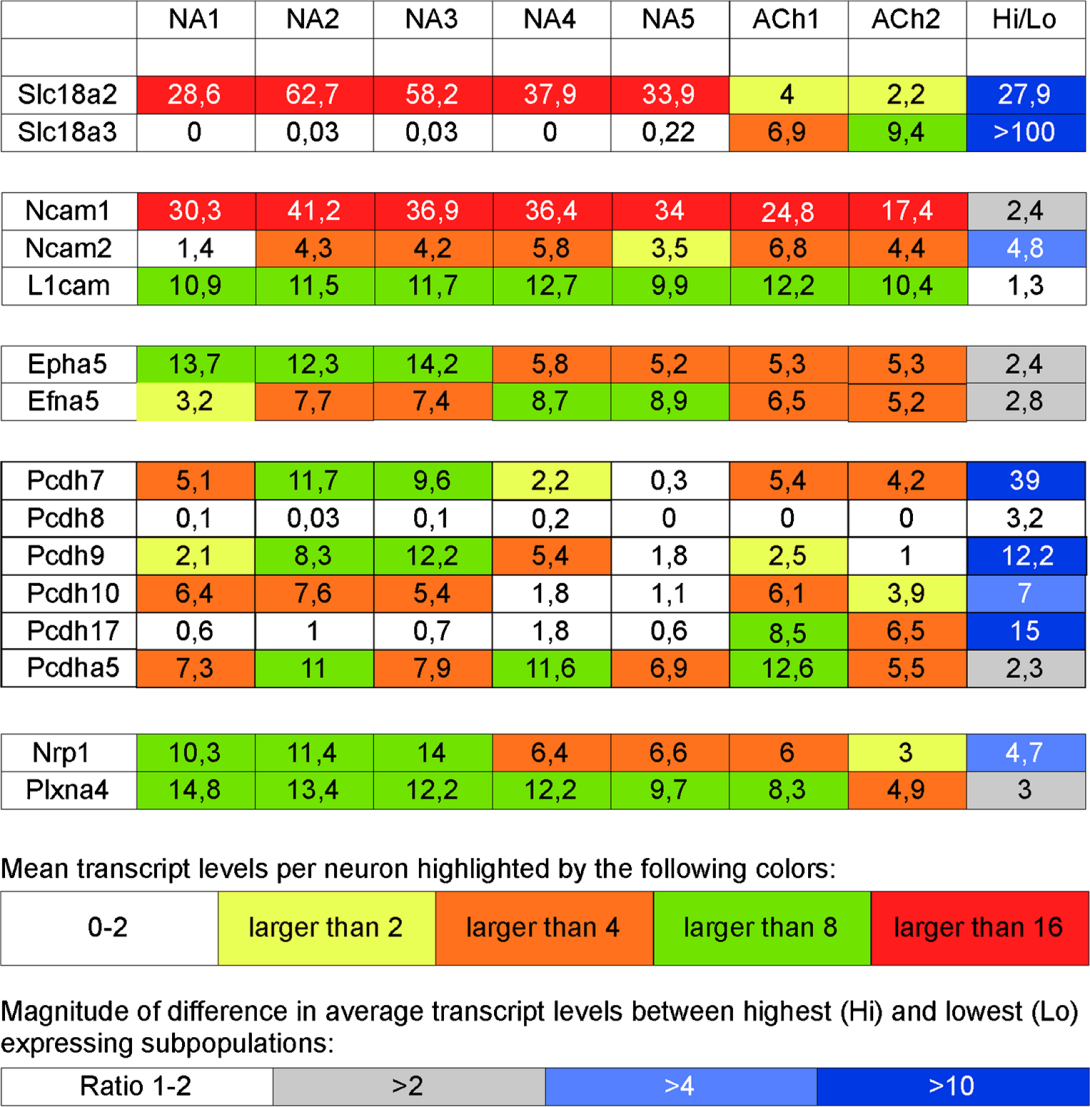
The expression of cell adhesion molecules, axonal outgrowth and synaptic organizer proteins in noradrenergic and cholinergic classes of mouse sympathetic neurons. The mean number of transcripts for the indicated genes are shown as determined by single cell RNA sequencing for the indicated populations of noradrenergic (NA1–5) and cholinergic (Ach1, 2) neurons from mouse thoracic sympathetic ganglia as provided by Furlan et al. (2016; supplementary table nn 4376-S4)

In particular, the expression of proteins involved in cell adhesion, contact formation, and synapse organization is of interest. Screening the data available for thoracic sympathetic neurons (Furlan et al. 2016) (Supplementary figure 4, nn4376) has provided first hints regarding the critical molecular players. Comparing the mean expression levels for a range of protein classes (Fig. 4) revealed only minor differences in the transcript expression levels between noradrenergic and cholinergic sympathetic neuron populations for the classical cell adhesion molecules Ncam1 and 2 and L1cam. Similarly, expression level differences in ephrins and their receptors were modest, including for the most highly expressed family members Efna5 and Epha5. More robust are the differences in transcript levels between sympathetic neuron classes for the semaphorin receptors, Neuropilin 1 and Plexin 4.

The most impressive differentially expressed transcripts that have been identified are those for selected protocadherin (Pcdhn) family members, which represent a group of proteins that have been recognized for their importance in multiple steps of neural circuit formation (Peek et al. 2017; Mountoufaris et al. 2018). The differences are comparable with those observed among the vesicular neurotransmitter transporters, which are the markers best suited for the characterization of noradrenergic and cholinergic neurons (Fig. 3). Transcript levels for Pcdh17 in cholinergic sympathetic neurons were, on average, 10-fold the levels observed in noradrenergic neurons. Comparable expression levels in noradrenergic neurons were detected for Pcdh 7, 9, 10 and Pcdha5. Importantly, the expression levels for the individual genes differ between the individual sympathetic neuron classes by up to 39-fold. In combination, these five protocadherin gene products seem sufficient to provide a chemical code specific for each sympathetic neuron class.

These findings indicate the need for an in-depth analysis of the role played by Pcdhns in the target-specific regulation of sympathetic effector functions, the establishment of target-specific neuronal pathways, and the developmental generation of neuronal class-specific Pcdhn gene expression patterns.

### Autonomic neurotransmitter plasticity in aging and degenerative processes: dysregulation of homeostatic control mechanisms

The stunning observation of the progression from mixed noradrenergic/cholinergic precursors to mature cells with segregated transmitter phenotypes during sympathetic neuron development was complemented by recent studies showing the expression of noradrenergic markers in mature cholinergic sympathetic neurons, as analyzed by RNA sequencing in mice (Furlan et al. 2016) and confocal imaging in humans (Donadio et al. 2019). Together with the changing prevalence of VIP-positive cholinergic stellate neurons in humans, from neonates and children to adults (Roudenok et al. 1999), and the increased expression of VIP in sympathetic neurons after acute myocardial infarction (Roudenok and Schmitt 2001), questions regarding the stability and plasticity of transmitter phenotypes during aging and disease processes represent key issues that must be addressed.

The detection of cholinergic transdifferentiation in the cardiac sympathetic nervous systems of humans and rodents during congestive heart failure and myocardial infarction, under the control of gp130-mediated mechanisms (Kanazawa et al. 2010; Olivas et al. 2016), demonstrates the depth to which developmental pathways that regulate autonomic neuron transmitter phenotypes can modulate the peripheral elements of autonomic control circuits in disease processes (Habecker et al. 2016).

High levels of sympathetic drive, associated with several cardiovascular diseases, including congestive myocardial infarction and hypertension, prompt the question for the component of sympathetic hyperactivity residing at the level of post-ganglionic neurons (Grassi et al. 2012; Shanks et al. 2013). A critical role in the pathogenesis of cardiovascular diseases is now recognized for NPY (Tan et al. 2018). Increased plasma levels of NPY and noradrenaline in patients with hypertension are considered indicators of enhanced sympathetic activity. In hypertensive rats, a developmental abnormality was observed in the numbers of NPY-positive cells, but differences in the amounts of TH and NPY IR in neuronal cell bodies were not detected (Gurusinghe et al. 1990, 1991). However, peripheral hyperinnervation is considered to be a major player in the functional changes observed in hypertensive rats (Head 1989). Hypertrophy of the neurons (Kondo et al. 1990) and their dendritic arbor (Peruzzi et al. 1991) have been reported in spontaneously hypertensive rats. Taken together, these results indicate the different activity statuses of postganglionic neurons, including increased transmitter and modulator release. The extent to which changes in the balance between noradrenergic and cholinergic phenotypes, and possibly between the nitrergic and purinergic properties of sympathetic neurons, affect disease states remains unclear.

Autonomic dysfunction constitutes a hallmark of a diverse range of neurodegenerative diseases, such as Parkinson’s disease and other synucleinopathies, diabetic neuropathies, and multiple sclerosis (Rafanelli et al. 2019). In addition, selective autonomic dysfunction may be associated with rare diseases, such as familial dysautonomia or pure autonomic dysfunction. Dysfunctions that affect autonomic homeostatic regulation can compromise cardiovascular and thermoregulatory control and the functions of the pelvic organs. Depending on the nature and severity of the disease, these functional changes may present with different time sequences, combinations, and intensities.

Advances in the clinical assessments of functional subsystems in the ANS have resulted from improved sudomotor testing, to complement the more conveniently recordable cardiovascular biosignals (Vinik et al. 2015; Ziemssen and Siepmann 2019). Quantitative results of sudomotor axon reflexes in Parkinson’s disease can be correlated with the presence of VIP in skin samples, to yield a measure of sweat gland activity and neuromodulator expression in the innervating sympathetic sudomotor neurons (Kawada et al. 2009). Comparably, sudomotor testing in diabetes allows correlations to be examined between peripheral autonomic fiber density, the degree of dysfunction (Gibbons et al. 2009; Krieger et al. 2018), and neuropeptide contents (Liu et al. 2015). Surprisingly, VIP levels in affected skin areas undergo an initial increase (Properzi et al. 1993), before ultimately declining (Levy et al. 1989, 1992). In multiple sclerosis, peripheral neuropathy has been associated with sudomotor dysfunction (Khan et al. 2018) and compromised sudomotor function has been correlated with impaired thermoregulatory sweating (Saari et al. 2008). Although disturbances in thermoregulation are known to constitute an important autonomic deficit in multiple sclerosis (Davis et al. 2010; Habek et al. 2016), IHC analyses of sweat gland innervation are currently lacking. In addition, studies of the cholinergic neurons in autonomic ganglia are lacking not only for multiple sclerosis but also for Parkinson’s disease and diabetes.

These studies exemplify approaches in which the neurotransmitter and neuromodulator features of post-ganglionic autonomic neurons are analyzed in the context of key autonomic dysfunctions and disease. This line of investigation has been pursued intensely in the human and rodent sympathetic nervous systems, in which changes in neuronal phenotypes, observed after myocardial infarctions, and alterations in peptidergic phenotypes in Parkinson’s disease and diabetes, mark particularly interesting cases. The human pelvic plexus, which has been less intensely studied along these lines, will be an even more challenging subject due to the extensive developmental regulation of neuronal phenotypes and the co-expression of noradrenergic, cholinergic, and nitrergic features. Pelvic floor dysfunctions make a particularly relevant example because of their age and gender-specific manifestations (Dieter et al. 2015). The expression of gender-specific differences in pelvic extramural and intramural neuron populations and their contributions to the diverse voiding and sexual dysfunctions observed in men and women have not been analyzed in sufficient detail, in humans. The significant changes in neuron population compositions associated with aging are also incompletely understood.

One subsystem associated with the autonomic innervation of pelvic organs, which has been characterized in some detail, is the innervation of the lower urinary tract (de Groat and Yoshimura 2015). Age-dependent alterations in the numbers of noradrenergic relative to cholinergic neurons have been observed for the intramural bladder ganglia, changing the balance between these two neurotransmitter pathways. The use of anticholinergic drugs for the treatment of overactive bladder among elderly individuals can tune these regulatory paths (Wagg 2018); however, the presence of numerous other neuromodulators in this system can also explain the insufficiency of this approach (Woodford 2018). Geriatric urinary incontinence (Ouslander 1992) is likely to remain an international problem (Searcy 2017), and the extent to which central and peripheral mechanisms contribute to stress incontinence (Yoshimura and Miyazato 2012), compared with geriatric urinary incontinence, should be explored.

### Summary and perspective

Taken together, these results have contributed to the increasing understanding of neuronal populations in the diverse autonomic ganglia including a highly refined knowledge regarding their transcriptomes. The transcriptomes provide quantitative data on axonal outgrowth and synaptic organizer molecules to indicate players involved in the formation of target specific homeostasis circuits. Insight into disease-associated plasticity of neuronal properties, and age-dependent transformations, has changed our understanding of the autonomic neuron pools and circuits that operate in homeostasis. In combination with improving clinical assessment instruments for analyzing autonomic dysfunction, these advances will greatly improve our insights into autonomic control and the disturbances that manifest during aging and disease. These advances need to be exploited for the development of personalized treatment strategies that consider age, gender, and disease duration of individual human patients affected by autonomic dysfunction and the underlying diseases.

## Abbreviations

CIL: Ciliary ganglion
SPG: Sphenopalatine ganglion
PTG: Corresponding to the human pterygopalatine ganglion
SMG: Submandibular ganglion
OG: Otic ganglion
SCG: Superior cervical ganglion
STG: Stellate ganglion
CG: Celiac ganglion
SMG: Superior mesenteric ganglion
IMG: Inferior mesenteric ganglia
APG: Anterior pelvic ganglion
MPG: Major pelvic ganglion
PP: Pelvic plexus
CA: Catecholamine
TH: Tyrosine hydroxylase
DBH: Dopamine beta-hydroxylase
ChAT: Choline acetyltransferase
VAChT: Vesicular acetylcholine transporter
NO: Nitrogen monoxide
NOS: Nitrogen monoxide synthase
VIP: Vasoactive intestinal peptide
NPY: Neuropeptide Y
SOM: Somatostatin
Pcdhn: Protocadherin
